# Titania Nanopores as Photoelectrocatalysts for Coupling Hydrogen Production with Plastic Reformation

**DOI:** 10.1002/advs.202509287

**Published:** 2025-07-28

**Authors:** Van Truc Ngo, Karan Gulati, Cheryl Suwen Law, Nguyen Que Huong Tran, Jingkai Lin, Damian L. Stachura, Andrew D. Abell, Huayang Zhang, Abel Santos

**Affiliations:** ^1^ School of Chemical Engineering The University of Adelaide Adelaide South Australia 5005 Australia; ^2^ Institute for Photonics and Advanced Sensing (IPAS) The University of Adelaide Adelaide South Australia 5005 Australia; ^3^ School of Dentistry The University of Queensland Herston QLD 4006 Australia; ^4^ Centre for Orofacial Regeneration Reconstruction and Rehabilitation (COR3) The University of Queensland Herston QLD 4006 Australia; ^5^ Department of Chemistry The University of Adelaide Adelaide South Australia 5005 Australia

**Keywords:** ethylene glycol oxidation, hydrogen production, metal oxide photoanode, PET reformation, photoelectrochemical

## Abstract

Photoelectrochemical (PEC) water splitting offers a sustainable pathway for solar‐to‐chemical energy conversion, yet its efficiency is often limited by sluggish water oxidation and the generation of low‐value oxygen. Here, the use of engineered titania nanopore (TNP) films is reported, fabricated via anodization and thermal annealing, as co‐catalyst‐free photoanodes for coupling hydrogen evolution reaction (HER) with polyethylene terephthalate (PET) reformation into high‐value formate. By tuning the crystallographic phase of TiO_2_ from amorphous to anatase and rutile, the optimized anatase‐phase electrode exhibits excellent PEC performance in a two‐electrode configuration, achieving a high steady‐state photocurrent density of [2.34 ± 0.67] mA cm^−2^, a hydrogen evolution output of 1771 ± 30 µL cm^−2^, a formate yield of 1.68 ± 0.05 mmol L^−1^, and a Faradaic efficiency of 85 ± 9.0%. Notably, despite the absence of noble metals or complex heterostructures, the PEC performance of the TNP films is comparable to, or even surpasses, that of reported systems employing additional co‐catalysts. This study establishes a simple and scalable PEC platform for simultaneous green hydrogen production and plastic waste valorization, offering new opportunities for sustainable energy and environmental technologies.

## Graphical abstract

This study establishes a simple and scalable photoelectrocatalyst platform for simultaneous green hydrogen production and plastic waste valorization, offering new opportunities for sustainable energy and environmental technologies.

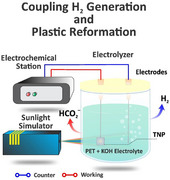

## 1 Introduction

Photoelectrochemical (PEC) water splitting is recognized as an innovative efficient method for clean storable energy sources (i.e., green hydrogen gas).^[^ ^1^, ^2^, ^3^, ^4^ ^]^ Unlike photocatalysis, PEC harnesses advanced engineered semiconductors to boost catalytic reactions driven by photoexcited electron–holes pairs under an externally applied potential bias. This applied bias facilitates charge carrier separation and suppresses rapid recombination, improving the overall reaction efficiency.^[^ ^5^, ^6^, ^7^, ^8^ ^]^ In conventional PEC water splitting systems, photoexcited electrons generated and migrated to the surface of semiconductors are rapidly driven to flow to the counter electrode upon the applied external potential bias, where the hydrogen evolution reaction (HER) occurs. Concurrently, the photoexcited holes participate in the oxygen evolution reaction (OER) on the catalyst (working electrode) to produce oxygen gas. Despite recent advances in semiconductor design, the overall catalytic efficiency of PEC water splitting remains limited by the sluggish kinetics of the anodic OER, which involves complex multi‐electron transfer processes.

Overcoming these kinetic barriers often requires applying a high overpotential, which unfortunately promotes the production of low‐value oxygen.^[^ ^9^ ^]^ Current efforts have focused on engineering anode materials for driving alternative oxidation reactions that are thermodynamically or kinetically more favorable than water oxidation.^[^ ^10^, ^11^, ^12^, ^13^, ^14^ ^]^ One promising option is to couple HER with the selective oxidation of waste‐derived organics to generate high‐value chemicals, such as formic acid. This new PEC route is devised as a promising dual‐benefit technology to enable systems for sustainable HER and PEC oxidation processes that simultaneously address both clean energy generation and environment challenges. State‐of‐the‐art advances in semiconductor materials have made it feasible to photoelectrochemically reform organic compounds into high value‐added chemicals in a controllable, efficient and chemically selective manner. Successful examples include the PEC reformation of glycerol into dihydroxyacetone organics, 5‐hydroxymethylfurfural into 2,5‐furandicarboxylic acid, and benzyl alcohol into benzaldehyde on highly catalytic BiVO_4_‐based composites.^[^ ^15^, ^16^, ^17^, ^18^, ^19^ ^]^ Current research efforts in this space have focused on using plastic waste sources instead of high‐purity plastic precursors to reform them into high value‐added chemicals to address the growing safety concerns associated with plastic pollution in the environment.^[^ ^20^, ^21^ ^]^ Of all plastic wastes, poly(ethylene terephthalate) (PET) has raised concerns because of its overuse and uncontrolled release in nature for decades. The strong C–C bonds in the chemical structure of PET and its high chemical resistance make it difficult to decompose and cleavage this plastic by natural means.^[^ ^22^, ^23^ ^]^ Therefore, treating PET waste by innovative means is of critical significance to address the environmental risk caused by the widespread use of this plastic. Existing PEC methods to reform PET require a pre‐treatment in heated alkaline electrolyte to form small organic compounds such as terephthalate (TA) and electrochemically active monomer ethylene glycol (EG),^[^ ^24^, ^25^ ^]^ where the latter compound enables PEC oxidation routes to generate formic acid/formate—a highly valuable chemical for industrial applications. However, to date, only a few studies have reported on the direct PEC‐driven reforming of PET.^[^ ^26^, ^27^ ^]^ Despite these advances, there remain key fundamental questions about the development of model PEC materials that can drive the coupling of HER–PET reformation in an efficient manner. Of all candidates, titania or titanium dioxide (TiO_2_) has a high catalytic activity, suitable band edges, superior photostability, non‐toxicity, and chemical stability, making it a highly attractive platform material for catalytic reactions.^[^ ^28^, ^29^ ^]^

In this context, this study explores the use of thin films composed of engineered titania nanopores (TNPs)—nanoporous TiO_2_ structures fabricated by electrochemical oxidation (anodization) of titanium (Ti)—as photoelectrocatalysts for the coupling of HER with high‐performing reformation of PET. The as‐produced TNP films were subjected to different temperatures (i.e., 400 and 900 °C) to tune the crystallographic phase of TiO_2_, increase active sites, and improve charge transfer and catalytic activity for enhancing PEC HER–PET reforming coupling performance. Comprehensive structural, optical, optoelectronic, and electrochemical characterizations were conducted to elucidate the properties of these model photoelectrocatalysts. PEC coupling of HER with PET reforming served as a model reaction to unravel enhancements associated with the combinational effect of the properties of these films. This study opens a pathway for the development of a new photoelectrocatalyst for HER–PET reforming, which could be extended to other plastic wastes and directly contribute to clean energy production, environmental applications and synthesis of high value‐added chemicals.

## 2 Results and Discussion

**Figure 1:**
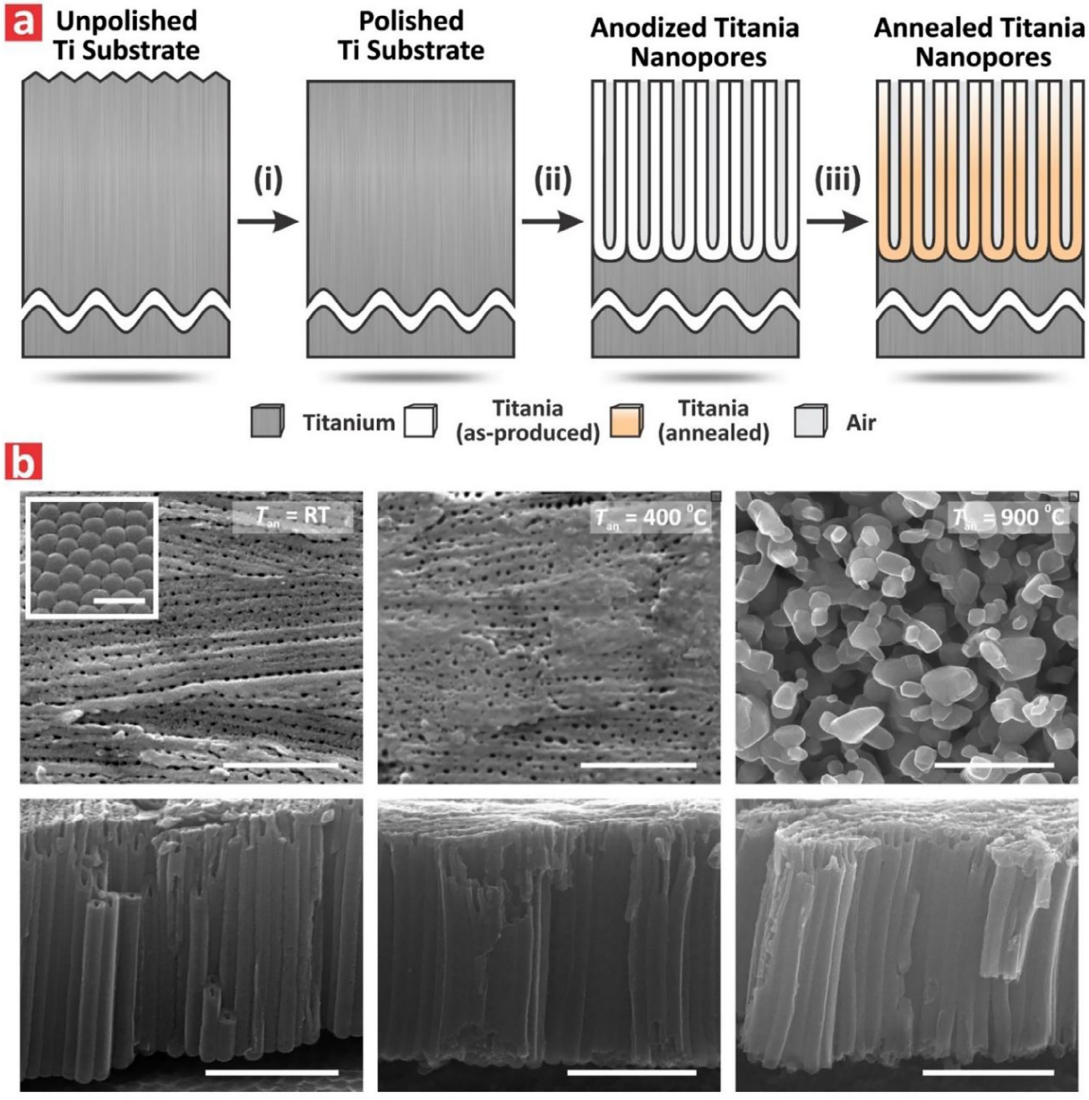
Structural engineering of annealed titania nanopores (TNPs). a) Fabrication steps with: (i) mechanical polishing of as‐received titanium substrates to obtain Ti substrates micro‐machined Ti surfaces, (ii) electrochemical oxidation (anodization) of polished Ti substrates to form TNPs films with a nanoporous TiO_2_ structure, and (iii) annealing of as‐produced TNPs to tune the crystallographic phase of TiO_2_. b) Top (top) and cross‐sectional (bottom) view FEG‐SEM images of TNPs produced at distinct annealing temperatures (i.e., *T*_an_ = room temperature (RT), 400, and 900 °C) with TNP_RT_ (left), TNP_400_ (center) and TNP_900_ (right) (NB: inset in TNP_RT_ (top) revealed the hemispherical barrier oxide layer at the bottom side of the film with scale bar = 250 nm; scale bars = 1 µm).

**Figure** **1**a depicts the fabrication process of TNPs by anodization of mechanically‐prepared micro‐rough titanium substrates in ethylene glycol‐based electrolyte.^[^ ^30^ ^]^ This process started with the mechanical polishing of as‐received Ti substrates to reduce the surface roughness and enable uniform nanopore growth during anodization (Figure 1ai). Next, polished Ti substrates were anodized in an appropriately aged electrolyte at 60 V for 10 min to form thin TNP films (Figure 1aii).^[^ ^31^ ^]^ The fabrication process concluded with an annealing step to adjust and transform the crystallographic phase of TiO_2_ forming the structure of the resultant TNPs (Figure 1aiii). Figure S1 (Supporting Information) shows a representative anodization profile that produces TNPs under potentiostatic conditions. The resultant TNP films were treated by thermal annealing at three temperatures (*T*_an_): room temperature (RT), 400, and 900 °C. These films were labelled as TNP_RT_, TNP_400_ and TNP_900_, respectively.

Figure 1b presents FEG‐SEM images of representative TNP_RT_, TNP_400_ and TNP_900_ films. It is apparent from these images that TNPs featured cylindrical nanopores that extended over the thickness of the film. The top and cross‐sectional view FEG‐SEM images of TNP_RT_ revealed that these structures had randomly distributed nanopores across all their surface, the average diameter of which was measured to be 41.8 ± 6.4 nm (Figure 1b, left). The length of the nanopores extended along the thickness of the film, from top to bottom, for a pore length of 1.8 ± 0.2 µm. The inset in Figure 1b revealed details of the hemispherical barrier oxide layer at the bottom side of the film. These FEG‐SEM images confirmed the successful and controllable fabrication of TNP_RT_ via anodization. Upon annealing at *T*_an_ = 400 °C for 2 h, the TNP_400_ film retained the original pore morphology of their TNP_RT_ counterparts, as revealed by FEG‐SEM image analysis (Figure 1b, center). The top view FEG‐SEM image showed the formation of a thin layer of amorphous–anatase TiO_2_ layer on some parts of the film, as confirmed by XRD analysis (vide infra).^[^ ^32^, ^33^ ^]^ Interestingly, FEG‐SEM image analysis of the resultant TNP_900_ films revealed more significant morphological changes in the films (Figure 1b, right). Top‐view FEG‐SEM images displayed the formation of crystal grains on the surface of the films, which were attributed to the formation of the rutile phase at *T*_an_ = 900 °C. However, the film integrity across its thickness was maintained after the thermal treatment, as indicated by the cross‐sectional FEG‐SEM images.

**Figure 2:**
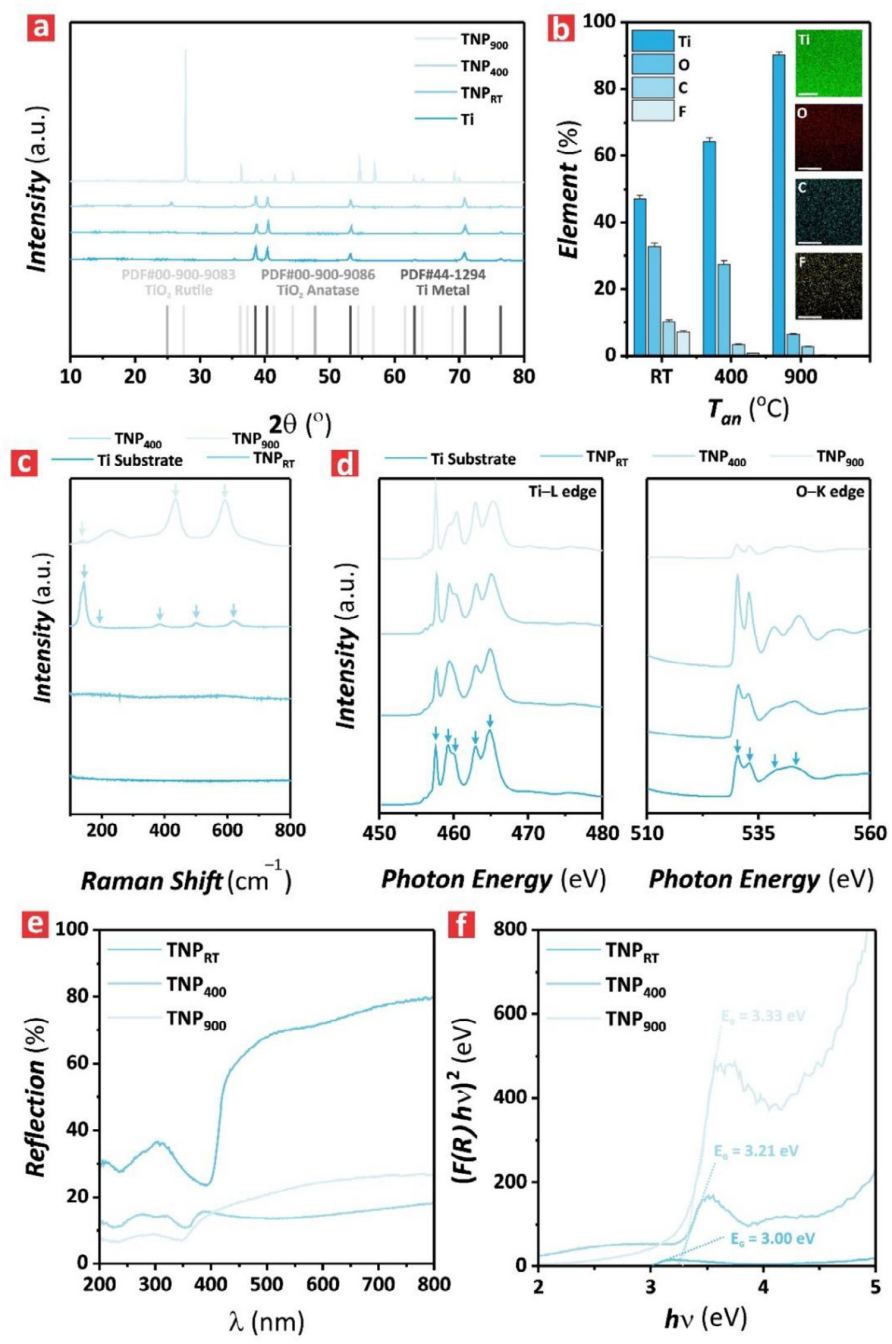
Chemical and crystallographic analysis of TNPs annealed at distinct temperatures. a) XRD spectra of representative Ti substrate, and TNP_RT_, TNP_400_ and TNP_900_ films with pair distribution function of Ti metal (PDF#44‐1294), anatase (PDF#00‐900‐9086) and rutile (PDF#00‐900‐9083). (b) EDX analysis of these films with a bar chart summarizing the elemental percentages of Ti, O, C, and F in TNPs fabricated at *T*_an_ = RT, 400, 900 °C (inset images show elemental mapping for Ti, O, C, and F in TNP_RT_, scale bars = 250 nm). c) Raman spectra of representative Ti substrate, and TNP_RT_, TNP_400_ and TNP_900_ films in the Raman shift range of 100–800 cm^−1^, where the different vibrational modes representing Ti–O and O–Ti–O bonds in annealed TNPs are denoted as A_1g_, E_g_, B_1g_ by arrowheads (i.e., from right to left: E_g_(v_1_), B_1g_(v_2_), A_1g_(v_3_), B_1g_(v_4_), E_g_(v_5_), and E_g_(v_6_) for TNP_400_, and, A_1g_, E_g_, B_1g_ and for TNP_900_). d) Soft X‐ray near‐edge absorption (XANES) spectra of representative Ti substrate, and TNP_RT_, TNP_400_ and TNP_900_ films with the Ti L‐edge (i.e., 450–480 eV) and the O K‐edge (i.e., 510–560 eV) peaks denoted from left to right as Ti_a_, Ti_b_, Ti_c_, Ti_d_, and Ti_e_, and O_a_, O_b_, O_c_, and O_d_ with arrowheads, respectively. e) Diffuse reflectance spectra of representative TNP_RT_, TNP_400_ and TNP_900_ films in the range of 200–800 nm. f) Estimation of electronic bandgaps (E_g_) of representative TNP_RT_, TNP_400_ and TNP_900_ films using the Kubelka–Munk function estimated from (e).

The crystallographic phase transformation of TNPs upon annealing was analyzed via XRD. **Figure** **2**a displays the XRD spectra of representative Ti substrate, TNP_RT_, TNP_400_ and TNP_900_ films, along with the pair distribution function (PDF) of Ti, TiO_2_ (anatase) and TiO_2_ (rutile). All spectra but that of TNP_900_ showed the characteristic diffraction peaks of Ti metal at 2*θ* ≈35.4, 38.6, 40.4, 53.3, 63.2, 70.8, 76.4, and 77.6°. This was associated with the underlying Ti substrate under the TNP films. Analysis of the XRD spectra of the TNP_RT_ film revealed the same characteristic diffraction peaks than those of the Ti substrate, indicating an amorphous TiO_2_ structure without any crystalline phase.^[^ ^34^, ^35^ ^]^ Upon annealing at *T*_an_ = 400 °C, TNP_400_ exhibited two characteristic diffraction peaks of crystallized TiO_2_ anatase phase at 2*θ* ≈25.6 and 48.3°, which were attributed to an amorphous‐to‐anatase phase transformation.^[^ ^36^, ^37^ ^]^ Further annealing at *T*_an_ = 900 °C induced crystallization of the TiO_2_ rutile phase, where the XRD spectrum of the TNP_900_ film featured characteristic diffraction peaks at 2*θ* ≈27.8, 36.4, 39.5, 41.6, 44.4, 54.7, 56.9, 64.5, 69.3, and 70.1°. Note that the characteristic peak of anatase at 25.8° was not present in the XRD spectrum of TNP_900_, which could be attributed to the complete anatase‐to‐rutile phase transformation at elevated temperature.^[^ ^38^, ^39^ ^]^ These results suggested that an annealing process facilitated the progressive crystallization of TiO_2_ in the structure of TNP films. Figure 2b presents a bar chart summarizing the effect of the annealing temperature on the elemental composition of TNPs, including EDX composition maps for Ti, O, C, and F in a representative TNP_RT_ film. The full EDX spectra of the TNP_RT_, TNP_400_ and TNP_900_ films are shown in Figure S2a—c (Supporting Information), respectively. Whereas Ti and O were associated with the TiO_2_ matrix forming the structure of the TNP films, small percentages of C and F were incorporated into the structure of TiO_2_ from the acid electrolyte during anodization. Upon thermal treatment, the relative percentage of Ti increased from 47.1 ± 1.1% to 90.1 ± 1.1% when the annealing temperature increased from *T*_an_ = RT to 900 °C. In contrast, the relative percentage of the other elements decreased swiftly during this treatment, with estimated values of 6.3 ± 0.5% for O, 2.8 ± 0.1% for C, and 0.2 ± 0.1% for F at *T*_an_ = 900 °C. Notably, fluorine is known to influence catalyst structure and surface properties. Prior studies have shown that residual fluorine can modulate the electronic structure, induce surface defects, and promote active site formation in transition metal fluorides, thereby enhancing oxidation reactions.^[^ ^39^ ^]^ It is therefore reasonable to assume that they may have some influence on the PEC activity of TNP films. These variations in elemental composition were attributed to the crystallographic phase transformation of TiO_2_ (i.e., reduction of O percentage), and the thermal oxidation of carbon and volatilization of fluoride upon extended high temperature treatment in air atmosphere.^[^ ^40^, ^41^, ^42^ ^]^ The formation of crystallized anatase and rutile phases upon the annealing was further confirmed by Raman analysis. Figure 2c summarizes the Raman spectra of Ti substrate, TNP_RT_, TNP_400_ and TNP_900_ for a Raman shift range of 100–800 cm^−1^.

The Ti substrate and TNP_RT_ film exhibited no characteristic Raman peaks, as it was expected for the pure metal form of Ti and the amorphous phase of TiO_2_.^[^ ^43^, ^44^ ^]^ In contrast, the TNP_400_ film featured six characteristic, well‐resolved peaks attributable to the presence of the anatase phase at 144, 195, 386, 502 and 621 cm^−1^, which correspond to six active vibrational modes (i.e., A_1g_, 2 B_1g_ and 3 E_g_) as denoted in Figure 2c. The Raman modes E_g_(*v*_1_), B_1g_(*v*_2_), and A_1g_(*v*_3_) corresponded to the stretching vibrations of Ti–O bonds in anatase TiO_2_. Due to the close proximity of the A_1g_(*v*_3_) and B_1g_(*v*_2_) modes, they merged into a single peak at 502 cm^−1^ when measured at room temperature. The modes of B_1g_(*v*_4_), E_g_(*v*_5_), and E_g_(*v*_6_) represented the bending type vibrations of O–Ti–O bonds in anatase.^[^ ^45^ ^]^ The Raman spectrum of the TNP_900_ film showed the four characteristic Raman peaks of rutile phase at 144, 436, and 593 cm^−1^, which corresponded to the vibrational modes B_1g_, E_g_, and A_1g_, respectively, and a broad peak at 229 cm^−1^. The formation of this broad peak was attributed to the second‐order scattering and disorder defects in the rutile phase.^[^ ^46^ ^]^

Figure 2d shows the soft X‐ray near‐edge absorption (XANES) spectra at the Ti L‐edge and O K‐edge for a representative Ti substrate, and TNP_RT_, TNP_400_ and TNP_900_ films, which provided crucial insights into the electronic configuration and bonding characteristics of these films. The Ti L‐edge spectrum (i.e., 450–480 eV) of these structures featured two characteristic peaks at L_3_ (≈458 eV) and L_2_ (≈463 eV), which were attributable to the spin‐orbit splitting of Ti 2p (i.e., Ti 2p_3/2,1/2_) core electrons as they transitioned into unoccupied Ti 3d states (i.e., Ti 3d_5/2,3/2_).^[^ ^47^ ^]^ The energy separation (i.e., ≈5 eV) between these peaks represented the characteristic of Ti^4^⁺ oxidation state in TiO_2_. The peaks exhibited greater sharpness and intensity with increasing annealing temperature, indicating enhanced crystallinity with fewer electronic defects in the structure of TiO_2_ with *T*_an_. The O K‐edge spectrum (i.e., 510–560 eV) presented noticeable pre‐edge peaks around 530–535 eV, which corresponded to O 1s → O 2p transitions hybridized with Ti 3d orbitals in the electronic structure of TiO_2_ (i.e., Ti 3d–O 2p hybridization). The increasing intensity of the peaks with *T*_an_ was observed from RT to 400 °C, suggesting stronger Ti–O orbital interactions in TNP_RT_ and TNP_400_ in comparison with those seen in TNP_900_. Further to that, the peaks located at 540–550 eV in the O K‐edge spectrum corresponded to the conduction band states primarily associated with Ti 4sp–O 2p hybridization (i.e., Ti 4s or Ti 4p hybridized with O 2p).^[^ ^48^, ^49^ ^]^ The intensity and shape of these peaks were observed in the O K‐edge of TNP_400_, suggesting a stronger overlapping of these orbitals in this form of TiO_2_ structure amongst its counterparts. The absence of notable energy shifts in the Ti L‐edge and O K‐edge spectra indicated the dominance of the Ti^4^⁺ oxidation state and a minimal presence of Ti^3^⁺ oxidation state in these TiO_2_ structures, suggesting a low concentration of oxygen vacancies. To further study the effect of annealing on the optoelectronic properties of these model semiconductor films, the diffuse reflection spectra of TNP_RT_, TNP_400_ and TNP_900_ were investigated (Figure 2e). All TNP films reflected light extensively in the visible–NIR light region (i.e., 400–800 nm wavelength), with 70% for TNP_RT_ and less than 30% for TNP_400_ and TNP_900_. Conversely, light reflection decreased significantly in the UV region to around 10% for TNP_400_ and TNP_900_, indicating that these two TiO_2_ structures absorbed UV light strongly. The estimation of the electronic bandgap (*E_g_*) for the TNPs using the Kubelka–Munk function (Figure 2f), revealed that the TNP_900_ film exhibited the widest electronic bandgap among its counterparts, with an estimated *E_g_* of ≈3.33 eV, followed by TNP_400_ with ≈3.21 eV and TNP_RT_ with ≈3.0 eV.

**Figure 3:**
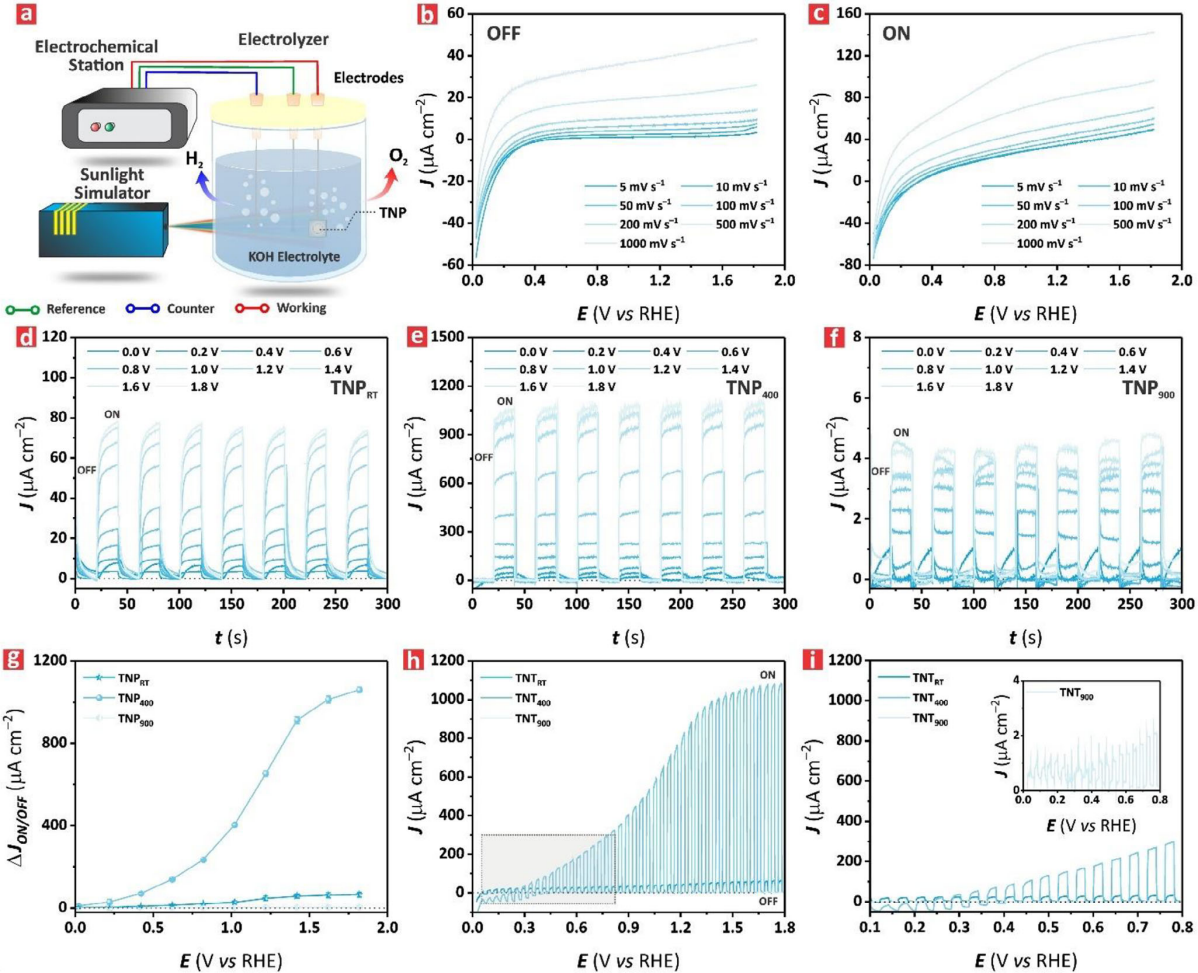
Electrochemical characterization of TNP films treated at different annealing temperature, from RT to 900 °C. a) Illustration of the three‐electrode system used to characterize the electrochemical and photoelectrochemical properties TNPs in 28 mL reactor containing 1.0 M KOH at pH 14 at 25 °C. b,c) Linear sweep voltammograms (LSVs) of TNP_RT_, TNP_400_ and TNP_900_ films at different scan rates (i.e., 5–1000 mV s^−1^) under varying overpotential (*E*), from 0.0 to 1.8 V versus RHE in 1.0 m KOH at pH 14 under b) OFF and c) ON illumination states, respectively. Chronoamperometry of d) TNP_RT,_ e) TNP_400_ and f) TNP_900_ under consecutive ON and OFF illumination states and varying *E*, from 0.0 to 1.8 V versus RHE, in 1.0 m KOH at pH 14 for a period of 20 s each and a total duration of 300 s. g) Summary of photocurrent density difference between ON and OFF illumination states (Δ*J*_ON/OFF_ = *J*_ON_‐J_OFF_) under varying *E* (i.e., 0.0, 0.2, 0.4, 0.6, 0.8, 1.0, 1.2, 1.4, 1.6 and 1.8 V versus RHE) obtained from (d–f). h) Linear sweep voltammograms of TNPs conducted at a scan rate of 1.0 mV s^−1^ in 1.0 m KOH at pH 14 under chopped light (i.e., short ON and OFF illumination periods of 20 s) under the range of *E* from 0.0 to 1.8 V versus RHE. i) Magnified view of linear sweep voltammograms of TNP_RT_, TNP_400_ and TNP_900_ films shown in (h) within the *E* range of 0.0–0.8 V versus RHE, with a transient delay at low applied potential range for the TNP_900_ film (inset).

**Figure** **3**a illustrates the three‐electrode system used to characterize the electrochemical and photoelectrochemical properties of TNPs in a reactor containing 28 mL of 1.0 m KOH solution at pH 14 and 25 °C. Figure 3b,c presents the current density–potential (*J–V*) characteristic of a representative TNP_RT_ under OFF and ON illumination modes under linear sweep voltammetry (LSV). The *J–V* curve revealed an increase of *J* with increasing potential (i.e., from 0.0 to 1.8 V vs RHE) and scan rate (i.e., from 5 to 1000 mV s^−1^) under both ON and OFF illumination states. The generated photocurrent increased more significantly at higher scan rates, with an estimated maximum value of 48 ± 2 and 141 ± 5 µA cm^−2^ at 1.8 V versus RHE at a scan rate of 1000 mV s^−1^ for the OFF and ON states, respectively. Similar trends of *J–V* characteristics were observed for the other TNP films (Figure S3, Supporting Information), revealing that TNP_400_ has the highest photocurrent with a maximum value of 1059 ± 15 µA cm^−2^, an approximate 60‐fold higher than that recorded for TNP_900_. A comparison of photocurrent density for different TiO_2_‐based systems in alkaline electrolyte conditions is summarized in Table S1 (Supporting Information).

Figure 3d–f presents the photoelectrochemical characterization of TNP films in terms of photocurrent density–time (*J*–*t*) characteristic at different overpotentials (*E*_OVP_) (i.e., 0.0, 0.2, 0.4, 0.8, 1.0, 1.2, 1.4, 1.6 and 1.8 V vs RHE). This analysis provided further insights into the dynamic behavior and efficiency of photoexcited charge separation and transport under illumination conditions. The *J–t* profile of the representative TNP_RT_ at *E*_OVP_ = 1.8 V versus RHE revealed that, under the ON illumination mode, *J* increased in a stepwise fashion, reaching a maximum average value of 78 ± 3 µA cm^−2^ for each 20 s pulse. When the light state was switched to OFF mode, *J* dropped dramatically to ≈0.0 µA cm^−2^ since the semiconductor was not capable of generating electron–hole pairs without external light excitation. This trend was consistent throughout the whole profile for the entire 300 s. Similar patterns were observed at other *E*_OVP_ values, demonstrating the responsivity and stability of TNP_RT_ under illumination over the range of *E*_OVP_ assessed. This behavior was also observed for the TNP_400_ and TNP_900_ films as shown in Figure 3e,f, respectively, with the highest *J* at all *E*_OVP_ achieved by the TNP_400_ film. Conversely, the TNP_900_ film demonstrated poor photoresponsive behavior, with the lowest value of *J* at any *E*_OVP_. The difference in photocurrent density between ON and OFF states, defined as *ΔJ* = *J*_ON_‐*J*_OFF_, was analyzed to gain further insights into this photoelectrochemical characteristic. *ΔJ* is a valuable figure of merit to quantify the capability of a given TNP film to generate electron–hole pairs, which is a critical aspect to consider to maximize photoelectrocatalytic reactions. Figure 3g illustrates the correlation between *ΔJ* and *E*_OVP_ for TNP_RT_, TNP_400_, and TNP_900_ films. This analysis indicated that TNP_400_ exhibited the highest *ΔJ* over the whole *E*_OVP_ range, reaching its maximum at a value of 1059 ± 13 µA cm^−2^ at *E*_OVP_ = 1.8 V versus RHE. This value was 263‐fold higher than that estimated for the TNP_900_ film, which achieved the minimum of *ΔJ* for the assessed TNP films. The enhanced performance of the TNP_400_ could be attributed to the crystallized anatase phase in its nanostructure, resulting in an enhancement in charge transport and electronic conductivity. Figure 3h shows the LSV conducted at a scan rate of 1.0 mV s^−1^ under chopped light conditions, revealing that *J* increased linearly with *E* for the TNP_RT_ and TNP_900_ films. In contrast, this parameter underwent an exponential increase for the TNP_400_ film.

**Figure 4:**
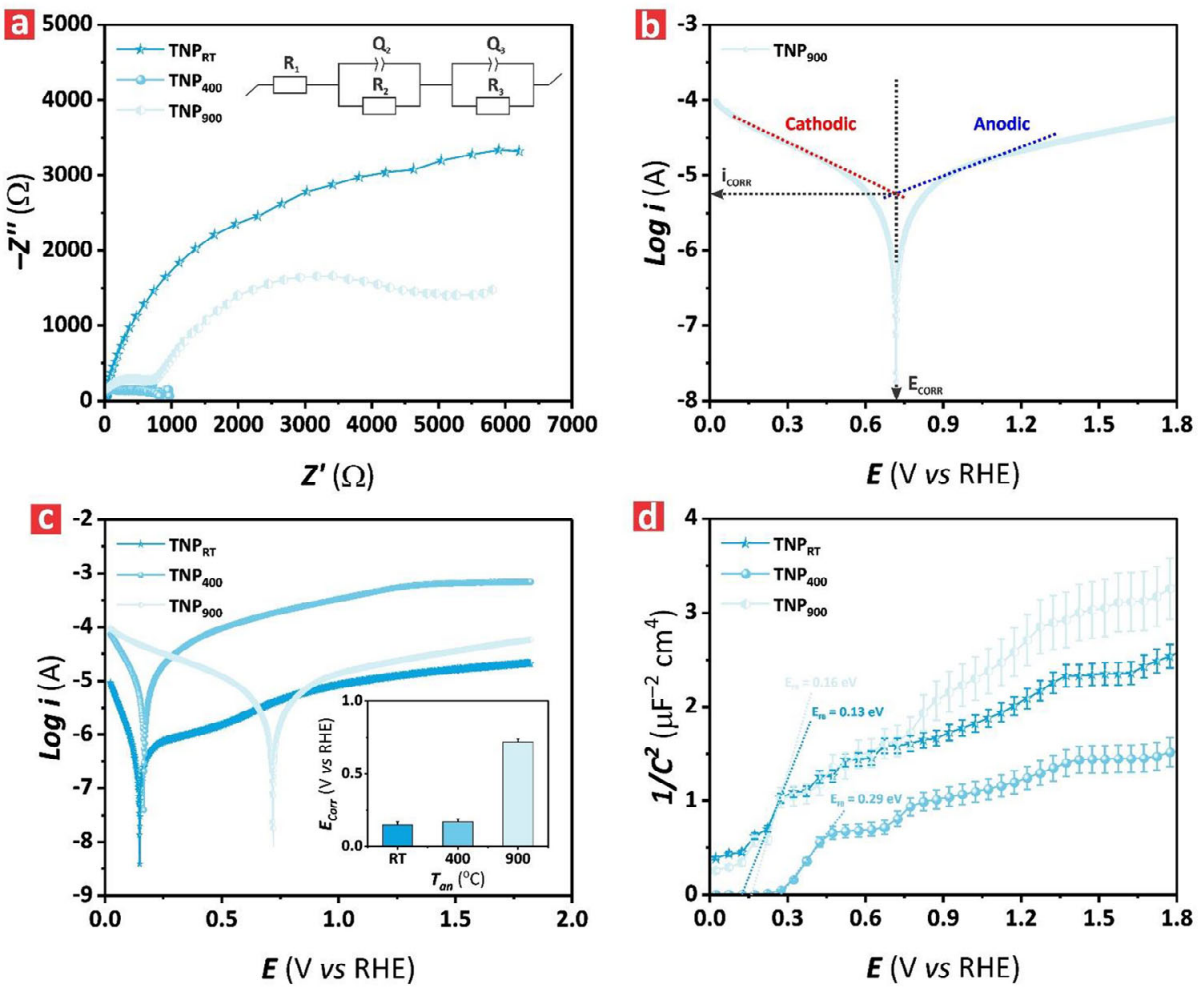
Electrochemical behavior of TNP films treated at different annealing temperatures, from RT to 900 °C. a) Electrochemical impedance spectroscopy (EIS) characterization of TNP_RT_, TNP_400_ and TNP_900_ at their corresponding open circuit potentials (*E_OCP_*) (i.e., 0.8, 0.76, 0.9 V vs RHE, respectively) within the frequency range of 1–10^5^ Hz in 1.0 m KOH at pH 14, and an inset illustrating the equivalent circuit composing of resistors (*R_1_, R_2_, R_3_*) and capacitors (*Q*_2_ *, Q*_3_) connected in series that provide a mechanistic description of the electrochemical behavior at the electrolyte–electrode interface. b) Representative Tafel plot with indication of estimation of corrosion current (*i*_CORR_) and potential (*E*_CORR_). c) Tafel plots of TNP_RT_, TNP_400_ and TNP_900_ conducted under the potential range of 0.0–1.8 V versus RHE in 1.0 m KOH at pH 14. d) Mott–Schottky graph (*C^−2^–E*) of TNP_RT_, TNP_400_ and TNP_900_ conducted under the potential range of 0.0–1.8 V versus RHE in 1.0 m KOH at pH 14 under a frequency of 1.0 kHz and OFF illumination state dark conditions, where dotted lines denote the flat‐band potentials (*E*_FB_).

A more detailed observation of the voltammogram at the range 0.0–0.8 V versus RHE in Figure 3i showed that *J* continuously increased with increasing *E* through the potential range for the TNP_RT_ and TNP_400_ films, while a transient decay was observed for the TNP_900_ film. This decay suggested a progressive recombination of photoexcited charge carriers, where not all photogenerated electrons could return to the back contact at low applied potential.^[^ ^50^ ^]^ **Figure** **4**a presents the electrochemical impedance spectroscopy (EIS) spectra of the TNP films at their open circuit potential (i.e., *E*_OCP_ = 0.8 V vs RHE). The Nyquist plots featured a semicircular shape across the specific frequency range of 1–10^5^ Hz. This analysis revealed that the TNP_400_ film showed the lowest *Z’* value of 1.2 ± 0.1 kΩ, featuring the smallest semicircle radius of all TNP films. This would suggest minimal charge transfer resistance and high electron–hole separation efficiency. Conversely, at low frequencies (i.e., 1.0 Hz) the TNP_RT_ and TNP_900_ films exhibited a high impedance, with a value of *Z’* of 6.1 ± 0.1 and 5.9 ± 0.1 kΩ, respectively, which denoted high charge transfer resistances at the electrode–electrolyte interfaces of these films. To investigate the effect of illumination on the charge transfer resistance in TNP films, we performed a comparative analysis using the electrochemical impedance spectra under ON and OFF illumination conditions (Figure S4, Supporting Information). The results revealed that all TNP films exhibited significantly larger impedance arcs under the OFF‐illumination state compared to those shown under illumination, indicating higher charge transfer resistance in the absence of photogenerated carriers. In particular, the TNP_400_ film demonstrated much smaller semicircle than that estimated for TNP_900_ and TNP_RT_ films. This is consistent with an improved charge transport mechanism. This analysis confirmed that illumination substantially reduced the interfacial charge transfer resistance, particularly for the TNP_400_ film, supporting its superior PEC performance. To gain further insights into the electrochemical behavior of the TNP films in 1.0 m KOH electrolyte, an equivalent circuit model was developed to fit the experimental data. This circuit consisted of electrical resistor (*R*) and capacitor (*Q*) components setup in series of (*R*_1_)‐(*Q*_2_//*R*_2_)‐(*Q*_3_//*R*_3_) (Figure 4a, inset), where *R*_1_ *, R*_2_ *, R*_3_ represented electrolyte resistance, charge transfer resistance and the TNP film resistance, respectively, and *Q_2_* and *Q_3_* were associated with the double layer capacitance and TNP film capacitance, respectively.^[^ ^51^, ^52^, ^53^ ^]^ The results revealed that the charge transfer resistance at the electrolyte–electrode interface of the TNP_400_ film was the smallest (i.e., 948 ± 16 Ω), which was nine‐fold lower than that estimated for the TNP_900_ film (i.e., 8436 ± 105 Ω).

This analysis was in good agreement with the interpretation of experimental EIS data analyzed in terms of *Z’* value. It was also found that the film resistance of the TNP_400_ and TNP_900_ films was 281 ± 8 and 482 ± 11 Ω, respectively—ten‐ and six‐fold lower than that estimated for the TNP_RT_ film. This result would suggest that the electrical conductivity increased with the annealing temperature and the transformation of amorphous TiO_2_ into high crystalline anatase and rutile phases, respectively. The electrochemical parameters obtained from the equivalent circuit and the fitting curves used to extract these are summarized in Table S2 (Supporting Information).

To study the corrosion behavior of the TNP films, we obtained the Tafel plots. Figure 4b shows the corrosion characteristics of current (*i*_CORR_) and potential (*E*_CORR_) for a representative TNP_900_ film obtained from its Tafel plot. The Tafel plots of all TNP films assessed in this study are summarized in Figure 4c, with an inset showing the correlation of the estimated corrosion potentials with the annealing temperatures. The results revealed that the TNP_900_ film exhibited a more positive *E*_CORR_ amongst its counterparts (i.e., 0.71 ± 0.05 V vs RHE), indicating that this TiO_2_‐based film was less prone to corrosion in 1.0 m KOH electrolyte because of the presence of the rutile phase in its structure.^[^ ^54^ ^]^ In contrast, the TNP_RT_ and TNP_400_ films showed less positive *E*_CORR_ (i.e., 0.15 ± 0.01 and 0.17 ± 0.01 V vs RHE, respectively) and were expected to be more susceptible to corrosion. These results were aligned with the quantified values of *i*_CORR_, which revealed that the TNP_400_ film deteriorated the fastest because of the high corrosion current (i.e., 10.8 ± 0.02 µA cm^−2^)—18‐fold higher than that for TNP_RT_ with a minimum *i*_CORR_ of 0.61 ± 0.05 µA cm^−2^. These findings indicated that the annealing process used in the TNP films had a significant impact on the corrosion behavior of these photoelectrocatalysts and that there was a direct correlation between corrosion resistance and the crystallographic phase of TiO_2_ films.

To further evaluate the electrochemical behavior at the semiconductor–electrolyte interface, we obtained the Mott–Schottky (*M–S*) diagram of the TNP films in 1.0 m KOH at 1.0 kHz under OFF illumination state (Figure 4d). The slopes of the linear curves from the *M–S* diagram were estimated to be positive, with values of 6.53 ± 0.06, 4.05 ± 0.05, and 8.87 ± 0.08, for TNP_RT_, TNP_400_ and TNP_900_ films, respectively, indicating that these TiO_2_‐based films were n‐type semiconductors. The flat‐band potentials (*E*_FB_) estimated from the M–S diagram were 0.13 ± 0.01, 0.29 ± 0.02, and 0.16 ± 0.01 V versus RHE for the TNP_RT_, TNP_400_, and TNP_900_ films, respectively. The positive shift of *E_FB_* indicated by the increase of *E*_FB_ with increasing annealing temperatures, from *T*_an_ = RT to 900 °C, denoted a downward shift in the Fermi level closer to the valence band within the electronic bandgap of the TNP_400_ and TNP_900_ films. Further to that, the charge carrier density, summarized in Table S3 (Supporting Information), was estimated to be the highest for TNP_400_ (i.e., 2.8 × 10^20^ cm^−3^) among its TNP film counterparts (i.e., approximately six‐fold higher than that estimated for the TNP_900_ film). These observations were consistent with the improved crystallinity of anatase and rutile phases and aligned with the superior PEC characteristics of the TNP_400_ film.

**Figure 5:**
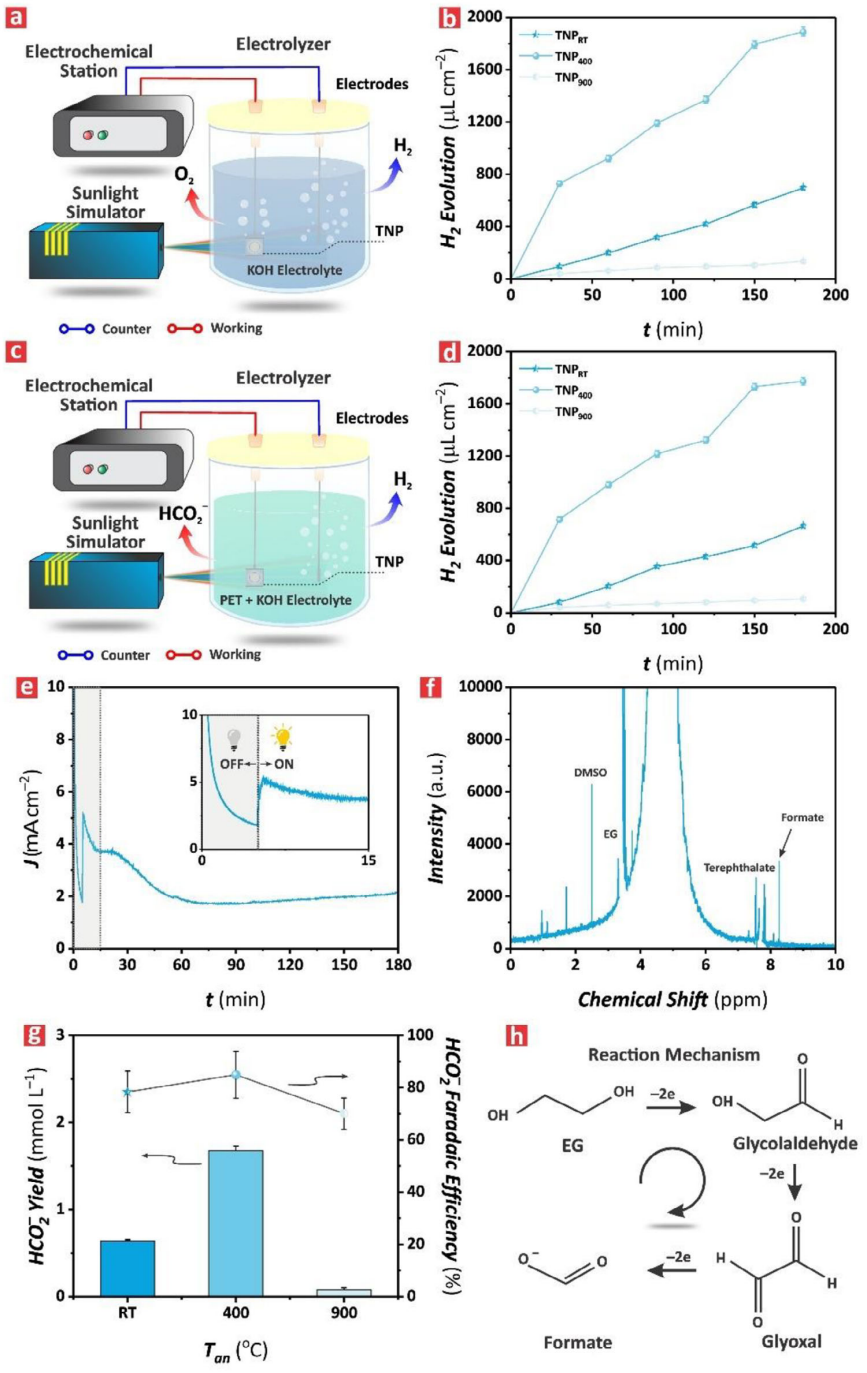
Coupling of HER with PET reformation in TNP films. a) Schematic of the two‐electrode system used to couple HER with PET reformation consisting of a counter electrode (i.e., Pt) and a working electrode (i.e., TNP films) in 2.0 m KOH electrolyte under the influence of an externally applied bias of 0.8 V under illumination conditions. b) PEC hydrogen evolution in terms of volume of H_2_ per unit area of TNP film with time (i.e., 30, 60, 90, 120, 150 and 180 min) in 2.0 m KOH electrolyte. c) Schematic of the two‐electrode system used to couple HER with PET reformation consisting of a counter electrode (i.e., Pt) and a working electrode (i.e., TNP films) in PET + 2.0 m KOH electrolyte under the influence of an externally applied bias of 0.8 V under illumination conditions. d) PEC hydrogen evolution in terms of volume of H_2_ per unit area of TNP film with time (i.e., 30, 60, 90, 120, 150 and 180 min) in PET + 2.0 m KOH electrolyte. e) A representative *J–t* diagram of the PEC process performed using a TNP_400_ film in PET+ 2.0 m KOH electrolyte operating at the external bias of 0.8 V, where the first 300 s of the reaction were performed under OFF illumination conditions and inset showing details of the initial 15 min of the reaction. f) A representative ^1^H NMR spectrum of the PET + 2.0 m KOH electrolyte upon exposure to the TNP_400_ film, where the distinctive peaks of formate (i.e., 8.29 ppm), dimethyl sulfoxide (DMSO) (i.e., 2.5 ppm), EG (i.e., 3.43 ppm), and aromatic protons of terephthalate (i.e., 7.87 and 7.64 ppm) are identified. g) Bar chart summarizing the formate yield (mmol L^−1^) and Faradaic efficiency (%) obtained from TNP_RT_, TNP_400_ and TNP_900_ films quantified from the coupling HER–PET reformation reactions performed in PET + 2.0 m KOH electrolyte. h) Proposed reaction mechanism of PET reformation into formate, involving two‐electron transfer oxidation processes, starting with EG and proceeding with the generation of intermediates such as glycolaldehyde and glyoxal.

A two‐electrode system was implemented to assess the performance of TNP films for HER–PET oxidation coupling. In this system, HER occurred at the counterelectrode (i.e., Pt electrode) whereas PET oxidation (i.e., conversion of EG into formate) was driven at the photoanode (i.e., TNP electrode) under illumination conditions. Experiments were conducted in two electrolytes: i) 2.0 m KOH and (ii) 25 mg mL^−1^ PET hydrolyzed in 2.0 m KOH (or PET + 2.0 m KOH). **Figure** **5**a illustrates the configuration of the two‐electrode PEC system used to perform the electrochemical characterization of the electrodes (i.e., Pt = counter; TNP_RT_, TNP_400_, and TNP_900_ films = photoanode) in 2.0 m KOH operated at an overpotential of 0.8 V. The corresponding *J*–*t* diagrams obtained from the TNP films are shown in Figure S5a‐c‐e (Supporting Information). The photocurrent density recorded for the TNP_400_ film rose swiftly after 300 s in the dark under the influence of the external bias potential and then declined steadily until reaching a plateau at an average value of 1780 ± 700 µA cm^−2^. This photocurrent density was two‐fold and nine‐fold higher than that for the TNP_RT_ and TNP_900_ films, respectively, suggesting that the TNP_400_ film achieved the best PEC activity of all its counterpart films. This result was consistent with the PEC properties established in previous characterizations. Figure 5b shows the H_2_ evolution reaction (HE) in terms of gas volume per unit area of photoelectrocatalysts for the TNP_RT_, TNP_400_, and TNP_900_ films under illumination at an overpotential of 0.8 V. This analysis revealed that HE increased progressively with time, where the performance obtained for the TNP_400_ film was 14‐ and 3‐fold higher than that recorded for its TNP_RT_ and TNP_900_ analogues, respectively. For example, at 180 min of irradiation the quantified volumes of H_2_ were 696 ± 15, 1891 ± 34, and 135 ± 10 µL cm^−2^ for the TNP_RT_, TNP_400_, and TNP_900_ films, respectively. The superior PEC performance of the TNP_400_ film was attributed to the presence of the TiO_2_ anatase phase in its nanostructure, resulting in a high electron–hole pair generation, an efficient separation of these charge carriers and the abundance of active reactive sites. Conversely, the TNP_900_ film featured a TiO_2_ rutile phase, which is known to have limited photoexcited charge generation and reduced charge transfer. As such, this model semiconductor film performed poorly compared to its counterpart films.

We further assessed the PEC performance of these model films using an electrolyte containing PET in 2.0 m KOH to couple HER with PET oxidation for the generation of high value‐added chemicals, such as formate (Figure 5c). The obtained results revealed a comparable pattern to that seen for the HE in 2.0 m KOH electrolyte but with the slightly lower generation of H_2_ volume (Figure 5d). For instance, the HE of the TNP_400_ film when running HER‐coupled with PET oxidation reached a maximum value of 1771 ± 30 µL cm^−2^ after 180 min irradiation. This reduction in H_2_ production could be attributed to slower hydrogen transport caused by electrolyte molecules building up on the counter electrode surface in PET + 2.0 m KOH.^[^ ^55^ ^]^ Interestingly, the analysis of the *J–t* diagram in these electrolytes revealed that, under the same overpotential and illumination conditions, the TNP electrodes achieved higher photocurrent current density in PET + 2.0 m KOH than that recorded in 2.0 m KOH. For example, the TNP_400_ film gave an average value of 2340 ± 670 µA cm^−2^ (Figure 5e). This would suggest a higher electrolyte conductivity and faster EG oxidation reaction (EGOR) on the surface of the TNP films compared to the sluggish water oxidation reaction in pure KOH. The *J–t* and HE diagrams in 2.0 m KOH and PET + 2.0 m KOH for the TNP_RT_, TNP_400_, and TNP_900_ films are shown in Figure S5 (Supporting Information) and a hydrogen calibration control is depicted in Figure S6 (Supporting Information). To evaluate the PEC reformation efficiency of PET into formate by TNP photoelectrocatalysts, the post‐reaction electrolyte was analyzed by ^1^H NMR spectroscopy. Figure 5f shows a representative ^1^H NMR spectrum of the electrolyte after oxidation by the TNP_400_ film. The presence of a characteristic formate peak at 8.29 ppm confirmed the successful conversion of EG to formate for all TNP electrodes, including TNP_RT_ and TNP_900_ (Figure S7, Supporting Information). A calibration curve for formate quantification was established beforehand (Figure S8, Supporting Information). The analysis of the ^1^H NMR spectra indicated that the TNP_400_ film achieved the highest formate yield (FY) at 1.68 ± 0.05 mmol L^−1^, which was 2.6‐ and 21‐fold higher than that for the TNP_RT_ and TNP_900_ films, respectively. These results were consistent with the analysis of the HE in 2.0 m KOH and PET + 2.0 m KOH electrolytes. The best performance shown by the TNP_400_ film over that of its TNP_RT_ and TNP_900_ film counterparts was attributed to three main properties. Firstly, better charge separation and transport properties: compared to the amorphous TNP_RT_ film, the TNP_400_ electrode showed significantly enhanced charge separation and transport properties. This was supported by its fast and stable response under chopped illumination conditions, the highest photocurrent density difference between ON and OFF illumination states (*ΔJ*) across a wide bias range, and the lowest charge transfer resistance observed from the EIS analysis. Conversely, the amorphous TNP_RT_ film suffered from extensive crystallographic disorder and charge trapping, which led to severe recombination losses. Although the TNP_900_ film was found to be highly crystalline, its rutile phase exhibited higher charge transfer resistance, lower Δ*J*, and a less favorable photoresponse, which is associated with larger grain sizes, reduced surface area, and less efficient carrier mobility. Second, increased number of active surface sites: the anatase‐phase TNP_400_ film features abundant catalytically active (101) facets, as confirmed by sharp and well‐defined diffraction peaks in the XRD spectra (2*θ* ≈ 25.6°) and a strong Raman band at 144 cm^−1^.^[^ ^56^, ^57^ ^]^ In contrast, no crystalline peaks were observed for the amorphous TNP_RT_, while TNP_900_ film showed broader, less intense signals, which is indicative of fewer reactive sites. XANES spectra further revealed that the TNP_400_ film featured stronger Ti–O coordination and fewer defect‐related states, suggesting improved active site stability and electronic structure. Lastly, a more suitable band structure: In terms of band energy, quantification of the electronic bandgap of these films revealed that TNP_400_ possessed a moderate bandgap of ≈3.21 eV, which was found to be wider than that of amorphous TNP_RT_ (≈3.00 eV) but narrower than that of rutile TNP_900_ (≈3.33 eV). Importantly, anatase has a more negative conduction band edge (−0.1 V vs NHE) than that of rutile (≈0.0 V vs NHE), which offers stronger reducing power for proton reduction in HER.^[^ ^58^ ^]^ This favorable band alignment contributes significantly to the superior performance of the anatase‐phase electrode.

Figure 5g presents the formate Faradaic efficiency (FE) for the TNP_RT_, TNP_400_ and TNP_900_ films—a critical figure of merit for validating the effectiveness of the EG conversion into formate. The TNP_400_ film exhibited the highest FE with an average value of 85 ± 9.0%, followed by the TNP_RT_ film at 78.4 ± 8.0% and the TNP_900_ film at 70.1 ± 6.0%. This result was well‐aligned with the PEC characteristics of these films, denoting the critical effect of annealing in enhancing the performance of the TNP films for the PEC reformation of PET. Notably, the TNP films in their pure form, without any functionalization with co‐catalysts (i.e., cobalt or nickel oxide) or precious metals (i.e., Pt, Pd) exhibited high FE and FY performances, which were comparable to those reported for more complex systems in the literature as summarized in Table S4 (Supporting Information).^[^ ^26^, ^59^ ^]^ This demonstrated the importance of tuning the annealing treatment to maximize the efficiency of this model photoelectrocatalyst. To further evaluate the solar‐to‐chemical conversion efficiency, the apparent bias photon‐to‐energy efficiency (ABPE) was estimated for the TNP_400_ film, which provided the best PEC‐driven PET reformation efficiency. The value of ABPE estimated was 0.13%, which is a reasonable value for a co‐catalyst‐free TiO_2_ photoanode.

In particular, annealing at 400 °C induced the crystallization of TiO_2_ anatase phase, which promoted the formation of active sites, which kinetically favored EGOR, a crucial step to achieve high‐performing photoelectrochemical reformation of PET into formate. Figure 5h illustrates the proposed mechanism for the photoelectrocatalytic reformation of PET by TNP films under illumination and external bias. In this process, EG, a primary product of PET hydrolysis in alkaline solution,^[^ ^60^ ^]^ undergoes oxidation on the porous TNP structure. Initially, EG is oxidized via two‐electron transfer to produce glycolaldehyde, an important intermediate. Glycolaldehyde is further oxidized to form glyoxal, which then undergoes oxidative C–C bond cleavage to generate formate.^[^ ^59^, ^61^ ^]^ It is important to note that the two‐electron transfer processes involved in EGOR proceed more readily than the complex four‐electron transfer required for water oxidation. Under an external bias and illumination, the efficient generation and separation of electron–hole pairs in the optimized TNP films promoted rapid reaction kinetics for the HER and EGOR, thereby significantly enhancing the overall PEC performance of the system.

To further evaluate the stability of TNP films under alkaline electrolyte conditions, we conducted a 48 h stability test for the TNP_400_ film, which is the best‐performing photoanode, in 2.0 m KOH + PET electrolyte under continuous illumination conditions at 0.8 V bias. The results shown in Figure S9 (Supporting Information) revealed that the photocurrent density initially decreased over the first 10 h to an average value of 1.95 ± 0.55 mA cm^−2^. Beyond this point, the photocurrent density remained relatively stable over the remaining 38 h. To evaluate structural integrity of the TNP_400_ film, we inspected the structure of the film after the stability test via FEG‐SEM imaging (Figure S10, Supporting Information). It was apparent from these images that the nanoporous structure of the TNP_400_ film remained largely intact, with only minor surface changes likely due to mild corrosion, which is expected for metal oxide photoelectrodes exposed to alkaline conditions over an extended period.

## 3 Conclusion

In this study, semiconductor films based on annealed titania nanopores were demonstrated as effective model photoanodes for coupling HER with PET reformation into formate without the need for co‐catalysts. Using a two‐electrode system configuration, operated at an external bias of 0.8 V and under illumination, the annealed TNP films achieved high performance, delivering a formate yield of 1.68 ± 0.05 mmol L^−1^ and a Faradaic efficiency of 85 ± 9.0%. The HER performance exhibited a high photocurrent density of [2.34 ± 0.67] mA cm^−2^, leading to a hydrogen evolution volume of 1771 ± 30 µL cm^−2^. These results revealed that coupling HER and PET reformation in a single PEC cell using a benchmark photoelectrocatalyst with engineered structural and crystallographic features is a promising strategy for simultaneous efficient conversion of plastic waste into high value‐added chemicals and generation of green hydrogen gas.

## 4 Experimental Section

### Chemicals and Materials

Titanium foils (99.5% purity, 0.25 mm thickness) were supplied by Nilaco (Japan). Ethanol (CH_3_CH_2_OH, EtOH), acetone ((CH_3_)_2_CO), ammonium fluoride (NH_4_F), ethylene glycol ((CH_2_OH)_2_, EG), dimethyl sulfoxide ((CH_3_)_2_SO, DMSO), deuterium oxide (D_2_O), and potassium formate (HCOOK) were purchased from Sigma–Aldrich (Sydney, Australia). Polyethylene terephthalate ((C_10_H_8_O_4_)_n_, PET), 200 mesh, molar mass of 150,000 g (g mol)^−1^, was supplied by Dongguan Zhangmutou Tesu Lang Chemical Raw Material Business Department (China). All aqueous solutions used in this study were prepared with Milli‐Q water (18.2 MΩ cm).

### Pretreatment of Plastic PET

To prepare the hydrolyzed PET electrolyte, 10 g of microplastic PET was dissolved in 400 mL of 2 m KOH. The solution was then magnetically stirred and kept at 70 °C in a convection oven for 48 h.

### Fabrication of TNPs by Anodization

Unpolished titanium foils were mechanically polished with decreasing sandpaper (i.e., 500, 800, and 1000 grits) to create a micro roughened surface with aligned microgrooves.^[^ ^31^ ^]^ The polished Ti foil was then cut into 1 × 1 cm squares, which were then cleaned by sequential sonication in acetone, ethanol, and Milli‐Q water before anodization. The samples were then anodized in an EG‐based aged electrolyte (i.e., 15 h anodization) containing 1% v/v water and 0.3% w/v NH_4_F in EG. Anodization was performed under a potential of 60 V for 600 s to form a thin film of TNP.^[^ ^30^, ^31^ ^]^ The fabricated TNPs were then washed thoroughly in Milli‐Q water several times, dried in an oven overnight and stored under dry conditions until further use.

### Preparation of Annealed TNPs

As‐produced TNPs were annealed at 400 and 900 °C in a Labec muffle furnace (Marrickville, Australia) at a heating rate of 10 °C min^−1^, and kept at these temperatures for 2 h in air atmosphere. Annealed TNPs were then cooled down to room temperature in the furnace. These samples were labelled as TNP_RT_, TNP_400_ and TNP_900_.

### Chemical and Structural Characterization of TNPs

The structure of TNP_RT_, TNP_400_ and TNP_900_ films was characterized by field emission gun scanning electron microscopy (FEG‐SEM Quanta 450, FEI, USA). The geometric features of these porous structures were determined by analyzing FEG‐SEM images using ImageJ software.^[^ ^62^ ^]^ The chemical composition and element mapping of TNP films were analyzed by energy‐dispersive X‐ray (EDX) spectroscopy during FEG‐SEM characterization. The crystallographic phases were characterized by X‐ray diffraction (XRD Rigaku MiniFlex 600, Japan).

### Optical Characterization of TNPs

The electronic bandgap of TNP_RT_, TNP_400_ and TNP_900_ films was estimated from diffuse reflectance spectra measurements characterized by a UV–visible spectrometer (UV‐2600, Shimadzu, USA). Raman spectra of Ti substrate, TNP_RT_, TNP_400_ and TNP_900_ films were characterized by a Raman confocal microscope (Lab RAM HR Evolution, Horiba, France) with laser excitation at 532 nm. The X‐ray absorption near‐edge structure (XANES) spectra of Ti substrate, TNP_RT_, TNP_400_ and TNP_900_ films for the O K‐edge and Ti L‐edge were obtained using the Soft X‐ray spectroscopy beamline at the Australian Synchrotron (Melbourne, Australia). All XANES experiments were conducted at room temperature under the conditions of ultra‐high vacuum. All the XANES spectra data were processed and analyzed using the QANT software program developed by the Australian Synchrotron.^[^ ^63^ ^]^

### Electrochemical and Photoelectrochemical Characterization of TNPs

The electrochemical and photoelectrochemical properties of TNP_RT_, TNP_400_ and TNP_900_ films were characterized in a setup comprising a custom‐built three‐electrode cell connected to an electrochemical workstation (CHI 760E Workstation, USA). The three‐electrode system consisted of a reference electrode (Ag/AgCl), a counter electrode (Pt), and the working electrode (i.e., TNP films). All the photoelectrochemical characterizations were conducted in an alkaline solution of 1.0 m KOH at pH 14 in the range of potential from ‐1.0 to 0.8 V versus Ag/AgCl. The measured potentials (vs Ag/AgCl) were converted to the reversible hydrogen electrode (vs RHE) using Equation 1:

(1) $$E_{\mathrm{RHE}}=E_{\mathrm{Ag}/\mathrm{AgCl}}+{E^{\theta }}_{\mathrm{Ag}/\mathrm{AgCl}}+0.0591\times \mathrm{pH}$$

where *E*_Ag/AgCl_ is the experimental measured potential and *E^θ^*_Ag/AgCl_ = 0.197 V (vs Ag/AgCl) at 25 °C.

The open circuit potential (*E*_OCP_) was measured for the TNP_RT_, TNP_400_ and TNP_900_ films. The current–potential (*I*–*V*) characteristics at ON and OFF illumination modes were measured at different scan rates, from 5 to 1000 mV s^−1^. The photocurrent–time (*J–t*) profiles were characterized at ten different potentials (i.e., from 0.0 to 1.8 V vs RHE) with an interval of 0.2 V, in the negative‐to‐positive bias direction under consecutive OFF and ON illumination modes for 20 s each until a total time of 300 s. The photocurrent density was then estimated to establish the *J–t* characteristic for a total functional area of 0.64 cm^2^. Electrochemical impedance spectroscopy (EIS) of these model films was characterized at *E*_OCP_ in the frequency range of 1–10^5^ Hz under illumination. The Tafel plot was characterized at a scan rate of 10 mV s^−1^. Mott–Schottky (M–S) curves were characterized at 10^3^ Hz and an amplitude of 5 mV s^−1^ under dark conditions. The flat‐band potential (*E*_FB_) of the photoanode was verified by measuring the capacitance of the space charge region (*C*) with an applied potential (*E*) through the M–S (Equation 2).

(2) $$\frac{1}{C^{2}}=\frac{2}{\varepsilon \varepsilon_{0}eA^{2}N_{d}}\;\times \left(E-E_{\mathrm{FB}}-\frac{K_{B}T}{e}\right)$$

where 𝜀 is the dielectric constant of the TNP films, 𝜀_0_ = 8.854 × 10^−12^ C V^−1^ m^−1^ (or F m^−1^) is the vacuum dielectric constant, 𝑒 = 1.60 × 10^−19^ C is the fundamental charge, *A* is the surface area of the photoanode, *N*_d_ is the carrier density, *E* and *E*_FB_ are the applied overpotential and flat band potentials, respectively, *K*_B_ = 1.38 × 10^−23^ J °K^−1^ is the Boltzmann constant, and *T* is the absolute temperature (°K). *N_d_* is combined with the M–S curve with Equation 3:

(3) $$N_{d}=\frac{2}{\varepsilon \varepsilon_{0}e}\;\times {\left[\frac{d\left(\frac{1}{C^{2}}\right)}{dE}\right]}^{-1}$$

where *C* is the space charge capacitance in the semiconductor, *E* is the bias applied on the electrode, $\frac{d(\frac{1}{C^{2}})}{dE}$ is the slope of the line.

### Assessment of Photoelectrocatalytic Performance and Formate Generation

The PEC performance of TNP films was characterized in a two‐electrode configuration system consisting of the Pt counter electrode and the TNP working electrode. The TNP films were mounted on the backside of a custom‐built reactor containing 28 mL of two types of electrolytes: i) 2.0 m KOH and ii) PET at 25 mg mL^−1^ hydrolyzed in 2.0 m KOH (i.e., PET + 2.0 m KOH). The reactant solution was magnetically stirred at 300 rpm in dark for 30 min to obtain an absorption–desorption equilibrium of electrolyte molecules adsorbed onto the surface of the TNP films. Argon gas was purged through the electrolyte for 30 min to remove dissolved oxygen. A Xenon lamp (PLS‐SXE300+/300UV Xenon Lamp Light Source, Perfectlight, China) with an optical power of ≈ 650 mW cm^−2^ was used as a simulated solar light for all our experiments, where the illumination was performed normally to the surface of the photoelectrocatalysts at a ≈15 cm distance. After 5 min operating at the bias potential of 0.8 V under dark conditions, illumination was applied for 180 min. H_2_ gas generated from HER driven by TNP films was quantified by gas chromatography with Argon as a carrier gas (8860 GC system, Agilent, Australia). After 30 min intervals of illumination, 500 µL of the generated gases was taken out of the reactor by a one‐mL gas syringe and then injected into the GC. Quantitative analysis of H_2_ generated in the photoelectrocatalytic reaction was estimated from the H2 area calibration curve obtained by quantifying the corresponding hydrogen peak area on the GC chromatograms from calibration controls using pure H_2_ gas. The degradation of micro‐plastic PET in the PEC reactions was determined by quantifying formate concentration in the reactant electrolyte via a nuclear magnetic resonance (NMR) spectroscopy. The ^1^H NMR spectra of electrolyte samples before and after PEC reactions were collected using a Varian Inova 600 MHz NMR. All NMR samples were prepared by mixing 0.5 mL of electrolyte with 0.1 mL of D_2_O, and 10 µL of aqueous DMSO (0.1% v DMSO in Milli‐Q water). The DMSO resonance (*δ* = 2.50 ppm) integral was used as the internal standard.^[^ ^59^ ^]^ Quantitative analysis was undertaken by first obtaining a calibration curve using a series of standard HCOOK solutions (i.e., 0.02, 0.05, 0.1, 0.4, 0.8, 1.0 mg mL^−1^) and the concentration of HCOOK in these experimental samples was quantified based on its resonance integral relative to the DMSO resonance integral.

The Faradaic efficiency (FE) of ethylene glycol oxidation and the yield of formate (FY) were calculated using Equations 4 and 5, respectively:

(4) $$\mathrm{FE}\%=100\%\times \frac{\mathrm{mole}\;\mathrm{of}\;\mathrm{produced}\;\mathrm{formate}}{\mathrm{total}\;\mathrm{charge}\;\mathrm{passed}\left(C\right)}\times \left(3\times 96485\frac{C}{\mathrm{mol}}\right)$$

(5) $$\mathrm{Formate}\;\mathrm{yield}\;\left(\frac{\mathrm{mmol}}{L}\right)=100\%\times \frac{\mathrm{mole}\;\mathrm{of}\;\mathrm{produced}\;\mathrm{formate}}{V\;\left(L\right)}$$

where 96485 C mol^−1^ is the Faraday constant, and *V* = 28  × 10^−3^ L is the volume of electrolyte used for the PEC measurements.

### Evaluation of Solar‐to‐Chemical Conversion Efficiency

As this PEC system operates under an applied external bias (0.8 V), the apparent bias photon‐to‐energy efficiency (ABPE) was calculated using the equation below^[^ ^64^, ^65^ ^]^

(6) $$\mathrm{ABPE}=\left[\frac{J_{P}\times \left(1.23-V_{\mathrm{bias}}\right)}{P_{\mathrm{in}}}\right]\times 100\%$$

where *J* is photocurrent density (mA cm^−2^), *V*_bias_ is an applied bias versus counter electrode (V), and *P*_in_ is the light intensity (mW cm^−2^).

## Conflict of Interest

The authors declare no conflict of interest.

## Supporting information



Supporting Information

## Data Availability

The data that support the findings of this study are available from the corresponding author upon reasonable request.
