# Supplementary material for: Titania Nanopores as Photoelectrocatalysts for Coupling Hydrogen Production with Plastic Reformation
Source: Adv Sci (Weinh). 2025 Jul 28;12(39):e09287. doi: 10.1002/advs.202509287 (PMC12533410; doi:10.1002/advs.202509287)
Supplement: Supplementary file 1 — Supporting Information [file ADVS-12-e09287-s001.pdf]

ADVANCED SCIENCE
Open Access

Supporting Information

for *Adv. Sci.*, DOI 10.1002/advs.202509287

Titania Nanopores as Photoelectrocatalysts for Coupling Hydrogen Production with Plastic Reformation

Van Truc Ngo, Karan Gulati, Cheryl Suwen Law, Nguyen Que Huong Tran, Jingkai Lin, Damian L. Stachura, Andrew D. Abell, Huayang Zhang* and Abel Santos**

Supporting Information

Titania Nanopores as Photoelectrocatalysts for Coupling Hydrogen Production with Plastic Reformation

Van Truc Ngo[1,2], Karan Gulati[3,4*], Cheryl Suwen Law[1,2], Nguyen Que Huong Tran[1,2], Jingkai Lin[1], Damian L. Stachura[2,5], Andrew D. Abell[2,5], Huayang Zhang[1,*], Abel Santos[1,2,*].

[1]School of Chemical Engineering, The University of Adelaide, Adelaide, South Australia 5005, Australia

*E-mail: k.gulati@uq.edu.au; huayang.zhang@adelaide.edu.au; abel.santos@adelaide.edu.au;

[2]Institute for Photonics and Advanced Sensing (IPAS), The University of Adelaide, Adelaide, South Australia 5005, Australia

[3]The University of Queensland, School of Dentistry, Herston QLD 4006, Australia

[4]Centre for Orofacial Regeneration, Reconstruction and Rehabilitation (COR3),The University of Queensland, Queensland, Herston QLD 4006, Australia

[5]Department of Chemistry, The University of Adelaide, Adelaide, South Australia 5005, Australia

WILEY-VCH

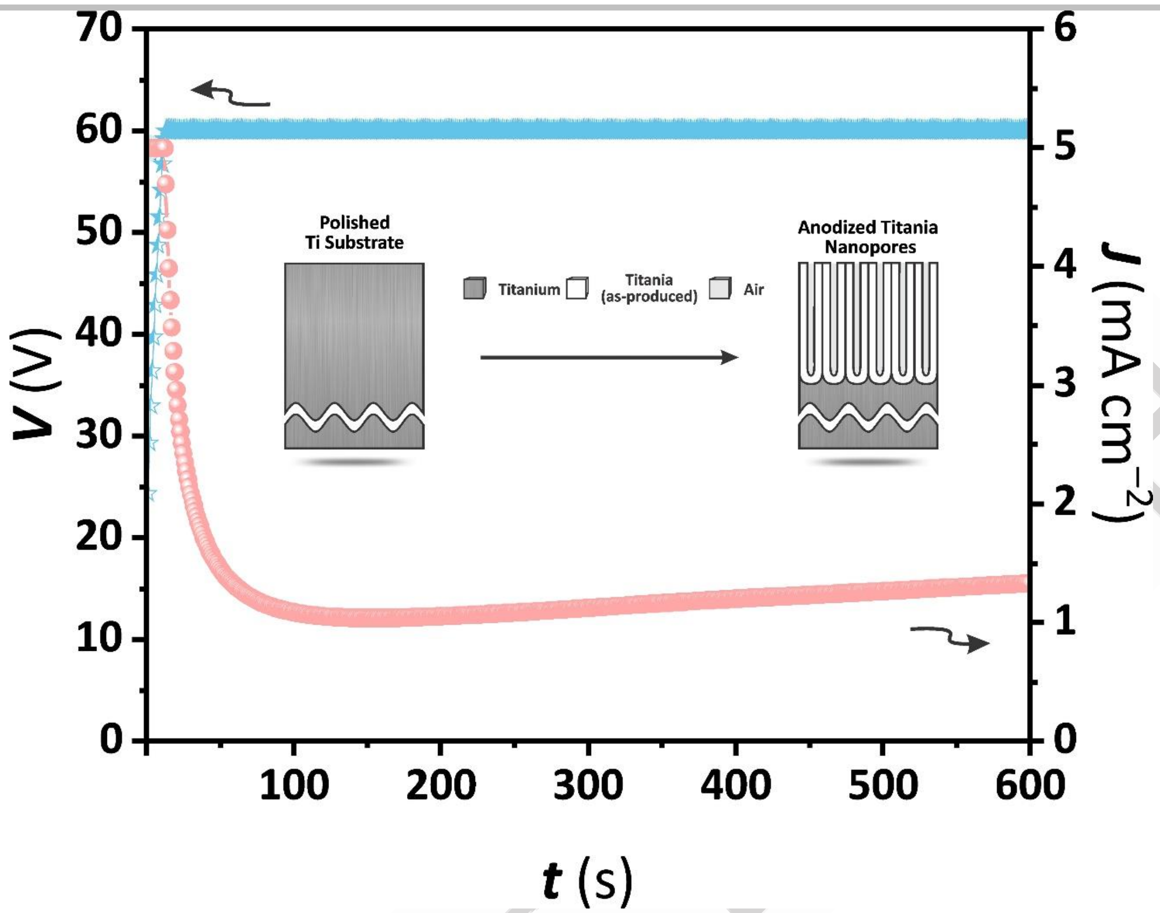

Figure S1. Representative electrochemical oxidation (anodization) profile (i.e., *V* and *J* vs *t*) used to fabricate titania nanopore films (TNPs), where the anodization process was performed over 15 h-aged EG-based electrolyte under potentiostatic conditions at 60 V for 600 s at room temperature. The current density output followed a characteristic pattern, where the sudden drop of current density during the first seconds of the anodization process denoted the formation of a thin film of anodic TiO_2 on the surface of the Ti substrate. As the anodization process continued, the nucleation and development of nanopores were denoted by a steady increase in current density with time. At this point, an electrochemical balance between the field-assisted formation and dissolution of anodic oxide at both sides of the barrier oxide layer closing the nanopore bottom tips in the TNP films were achieved.

WILEY

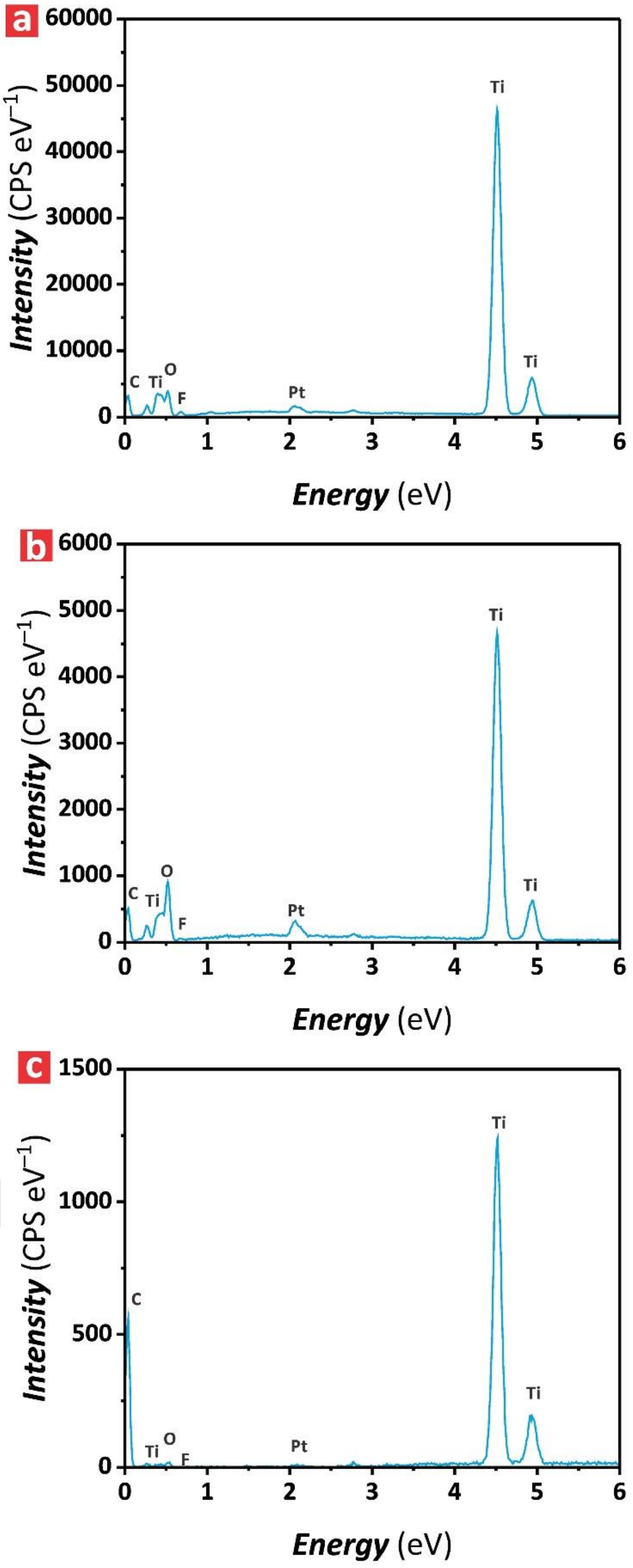

Figure S2. EDX spectra of annealed TNP films: (a) TNP_{RT}, (b) TNP_{400}, and (c) TNP_{900}. All EDX spectra showed the presence of four main elements including Ti, O, C, and F, where Ti and O were associated with Ti and TiO_2 from the Ti substrate and TNP film, respectively, C and F from from the anodizing electrolyte. Pt was attributed to the conductive coating used for FEG-SEM imaging.

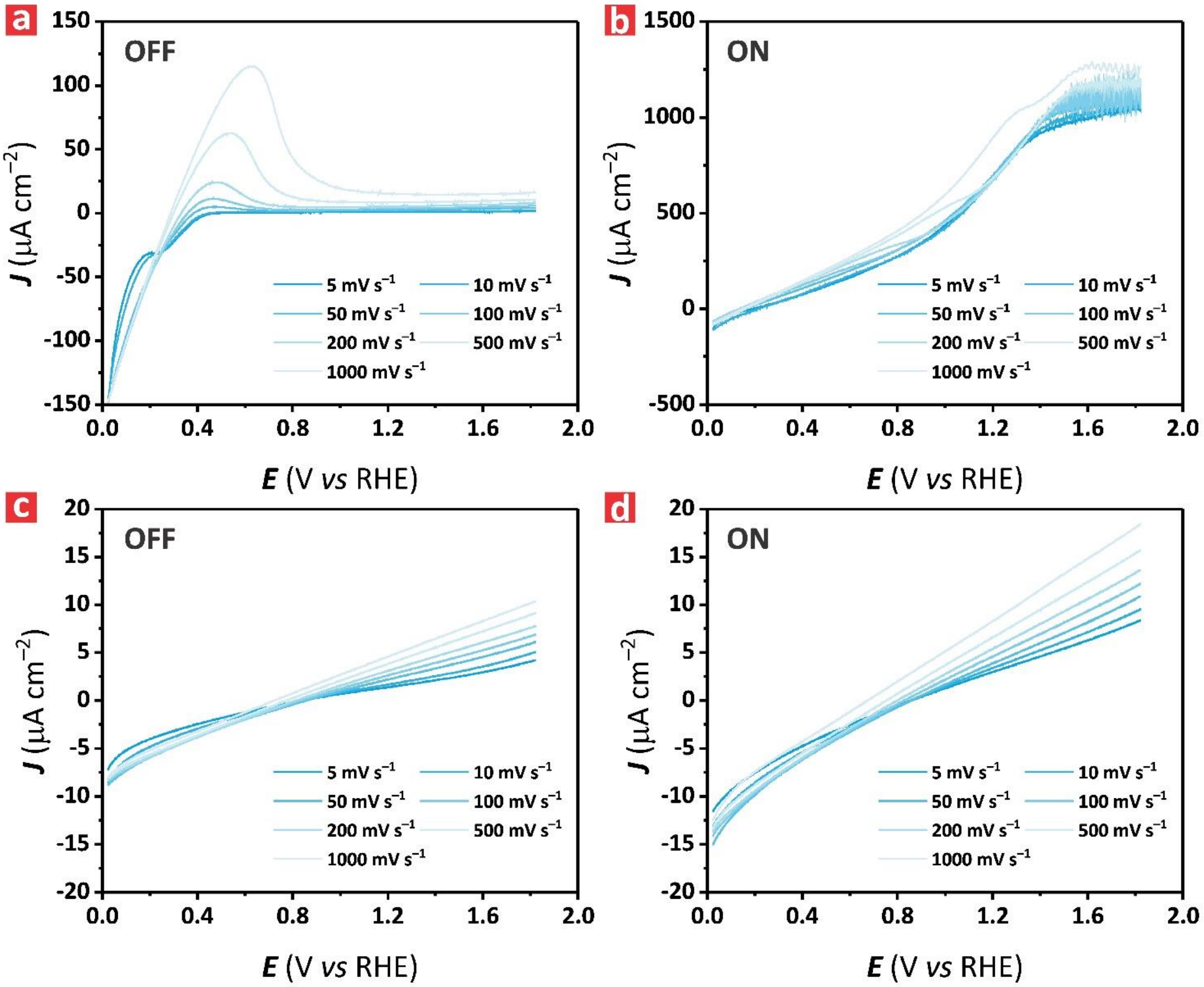

Figure S3. Electrochemical characterization of TNP films treated at different annealing temperature, from RT to 900°C. (a and b) Linear sweep voltammograms at OFF and ON illumination modes for the TNP_{400} film at 0.0–1.8 V *vs* RHE at different scan rate, from 5 to 1000 mV s^{-1}. (c and d) Linear sweep voltammograms at OFF and ON illumination modes for the TNP_{900} film at 0.0–1.8 V *vs* RHE at different scan rate, from 5 to 1000 mV s^{-1}. All measurements were conducted in 1.0 M KOH at pH 14 electrolyte.

WILEY

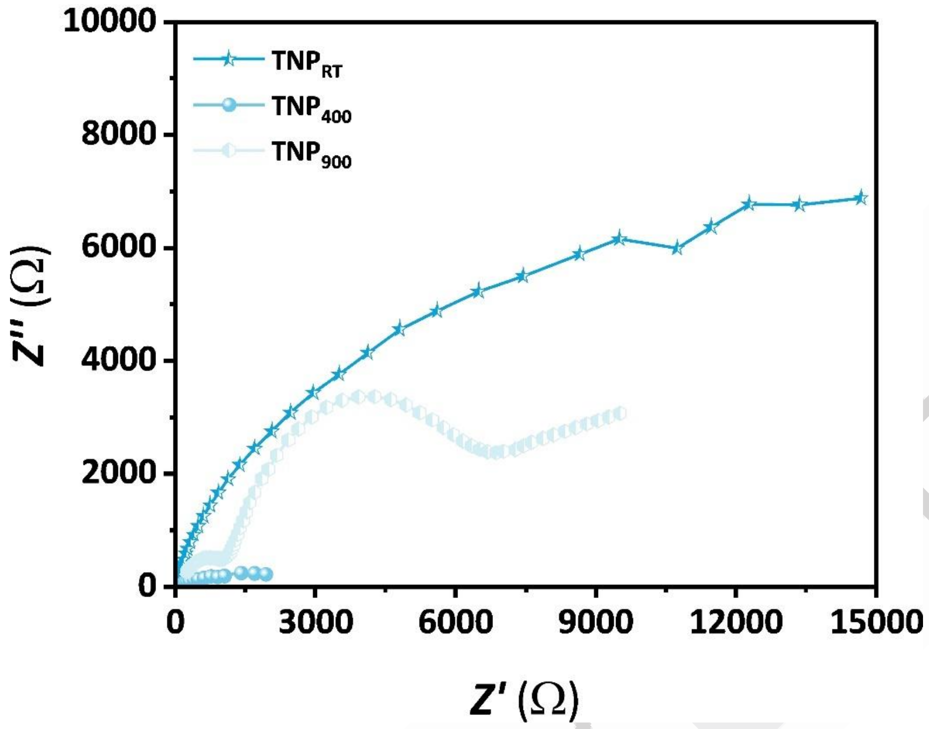

Figure S4. Electrochemical impedance spectroscopy (EIS) spectra of TNP_{RT}, TNP_{400} and TNP_{900} films at their open circuit potential under dark conditions (i.e., OFF illumination state) in 1.0 M KOH electrolyte.

WILEY-VCH

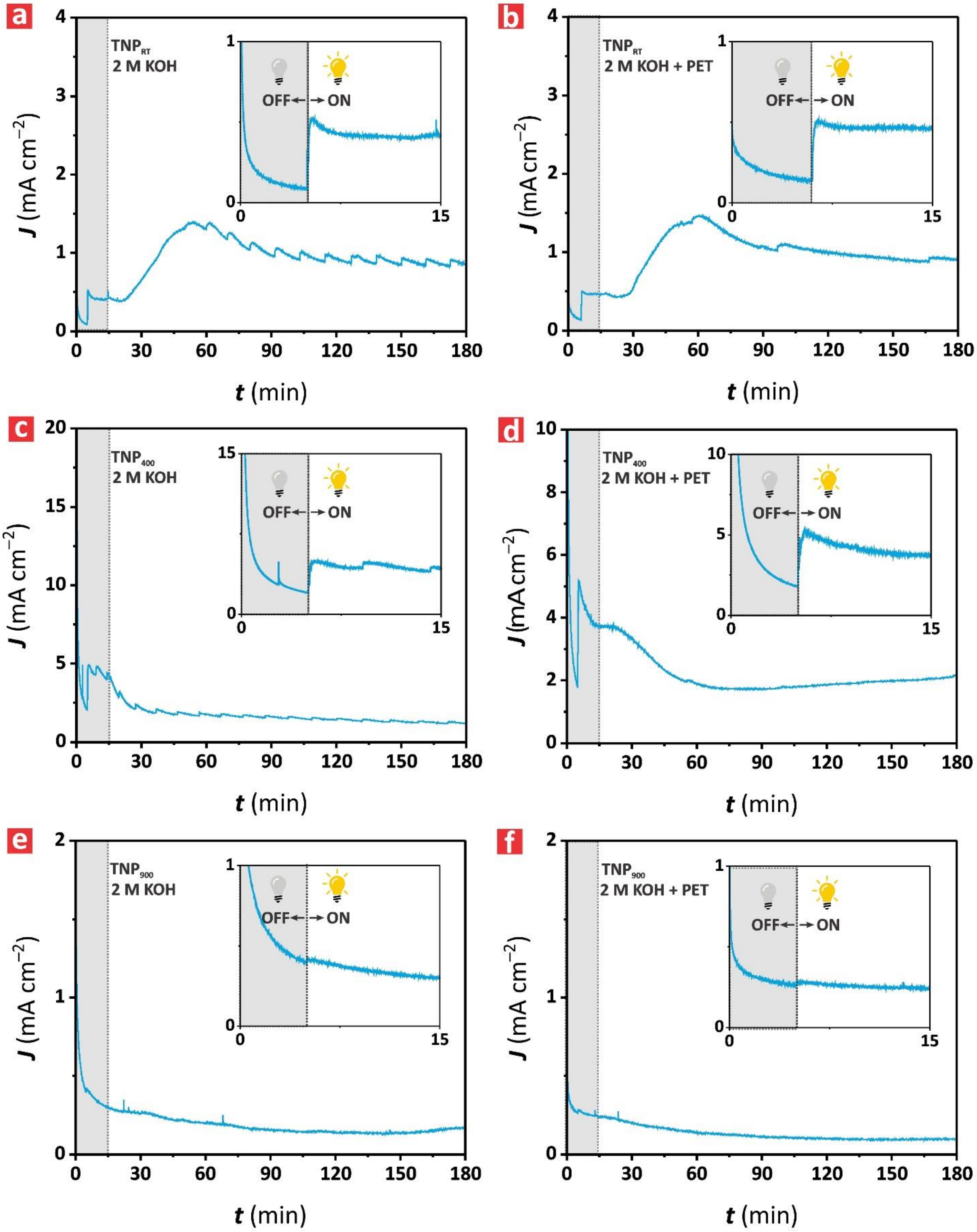

Figure S5. Photoelectrochemical characterization of HE and HER–PET reforming coupling in TNP films. (a, c and e) *J–t* diagrams of the PEC process performed using TNP_{RT}, TNP_{400} and TNP_{900} films in 2.0 M KOH electrolyte operating at the external bias of 0.8 V, respectively, where the first 300 s of the reaction were performed under OFF illumination conditions and inset showing details of the initial 15 min of the reaction. (b, d and f) *J–t* diagrams of the PEC process performed using TNP_{RT}, TNP_{400} and TNP_{900} films in PET + 2.0 M KOH electrolyte operating at the external bias of 0.8 V, respectively, where the first 300 s of the reaction were performed under OFF illumination conditions and inset showing details of the initial 15 min of the reaction.

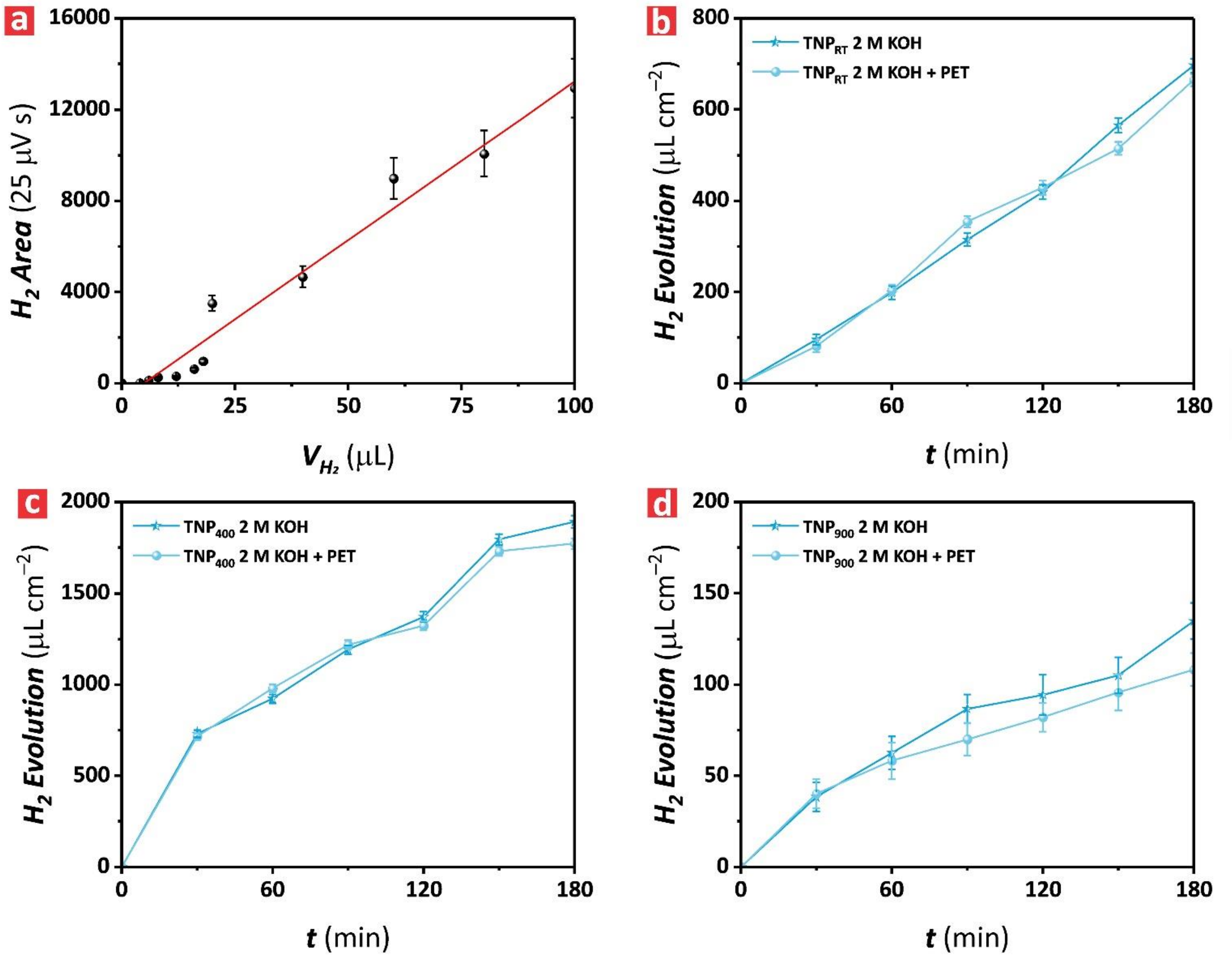

Figure S6. Quantification of H_2 gas generation from TNP_{RT}, TNP_{400} and TNP_{900} films in 2.0 M KOH and 2.0 KOH + PET electrolytes operating at the external bias of 0.8 V and under illumination. (a) Linear correlation between H_2 peak area and hydrogen volume (V_{H2} (µL)) in PEC reactions, where the H_2 peak area was 1139 *[V_{H2}]* – 681 and the R^2 value for the calibration line was 0.9693. (b–d) Comparative PEC hydrogen evolution in terms of volume of H_2 per unit area of TNP films with time (i.e., 30, 60, 90, 120, 150 and 180 min) for TNP_{RT}, TNP_{400} and TNP_{900} films, respectively, in 2.0 M KOH and PET+ 2.0 M KOH electrolytes.

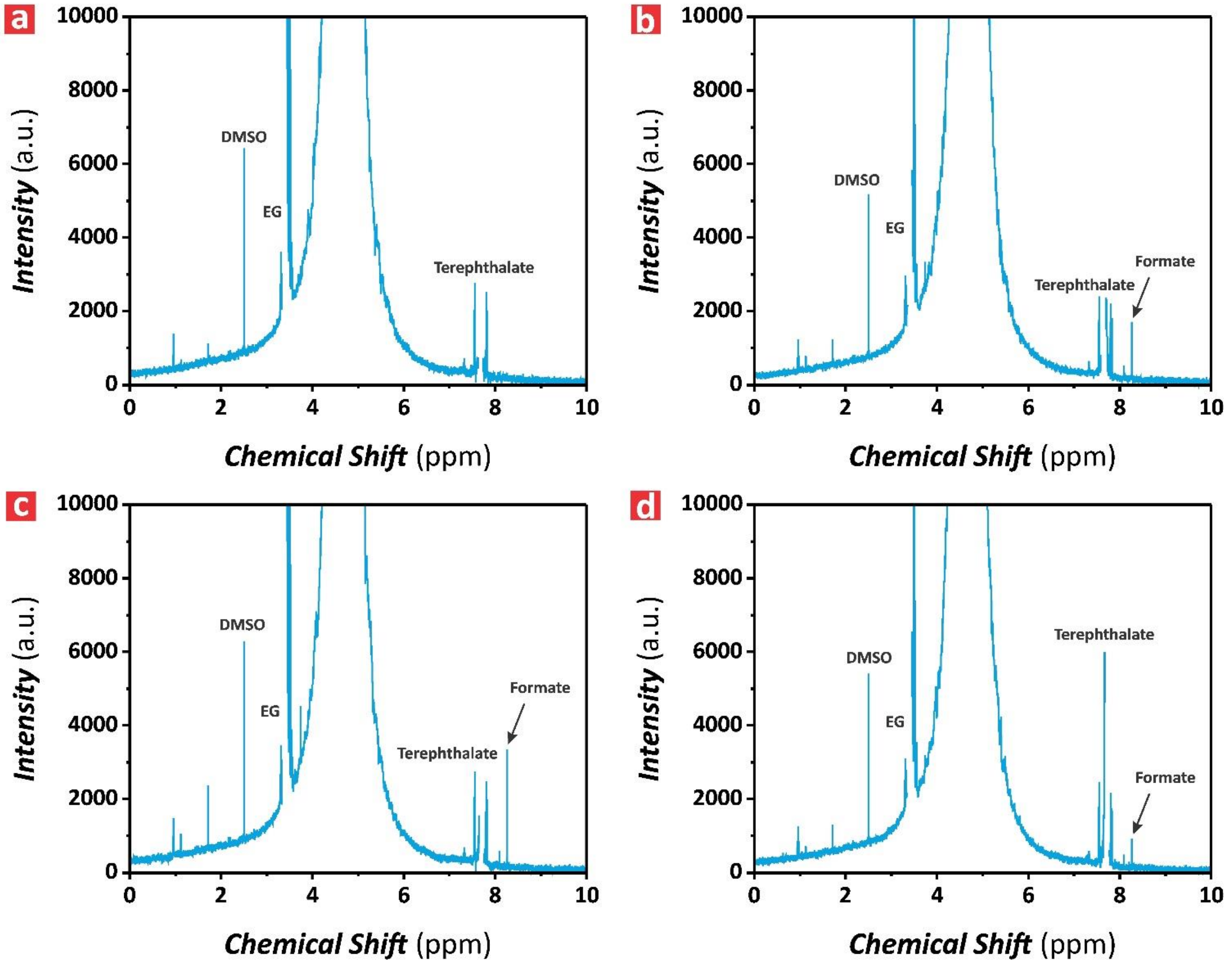

Figure S7. PET reformation characterization. (a) Representative 1^H NMR spectrum of the as-produced PET + 2.0 M KOH electrolyte, where the distinctive peaks of dimethyl sulfoxide (DMSO) (i.e., 2.5 ppm), EG (i.e., 3.43 ppm), and aromatic protons of terephthalate (i.e., 7.87 and 7.64 ppm) are identified. (b) representative 1^H NMR spectrum of the PET + 2.0 M KOH electrolyte upon exposure to the TNP_{RT} film. (c) representative 1^H NMR spectrum of the PET + 2.0 M KOH electrolyte upon exposure to the TNP_{400} film. (d) representative 1^H NMR spectrum of the PET + 2.0 M KOH electrolyte upon exposure to the TNP_{900} film. All these spectra exhibited the distinctive peak of formate at 8.29 ppm.

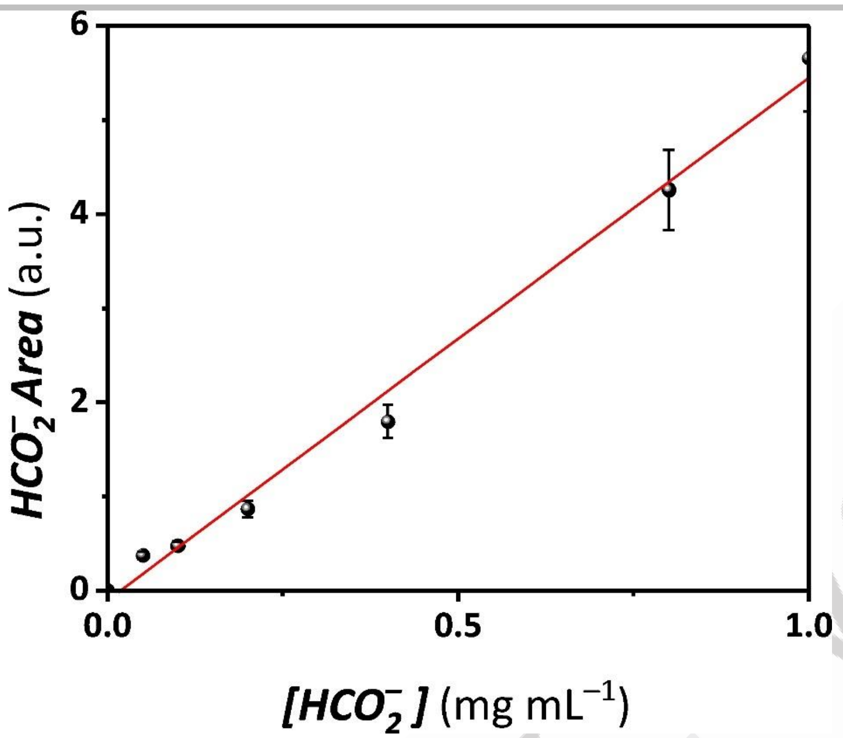

Figure S8. Linear correlation between formate concentration and formate integrated area. The fitting line for formate (HCO^{2-}) was HCO^{2-} area = 5.553 [HCO^{2-}] – 0.10175, where HCO^{2-} area has the arbitrary unit (a.u.) and [HCO^{2-}] is the formate concentration (mg mL^{-1}). The R^2 value for the calibration line was 0.99063.

WILEY-VCH

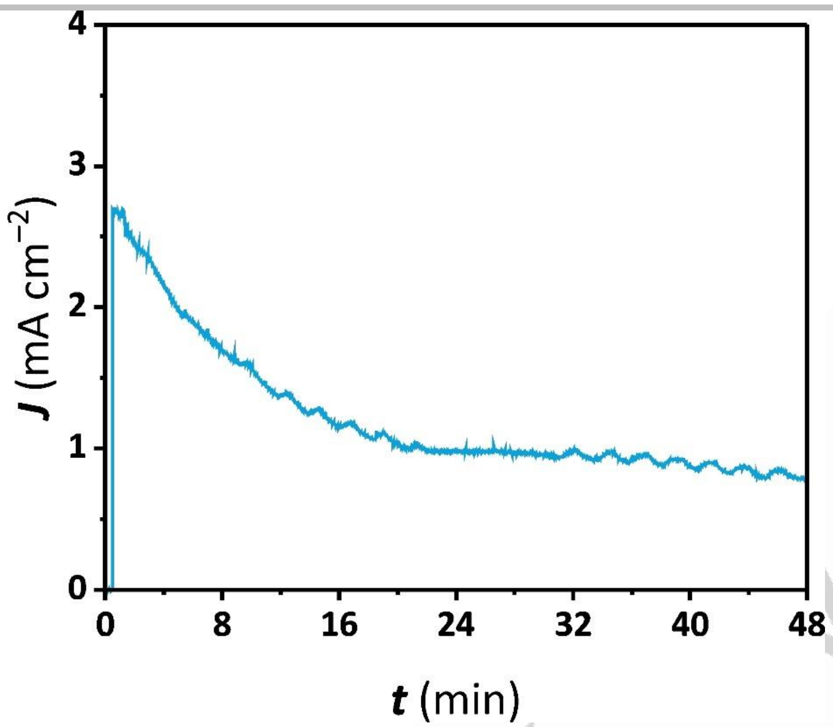

Figure S9. 48-hour stability test performed over the TNP_{400} film under illumination at the bias voltage of 0.8 V in 2.0 M KOH + PET electrolyte (NB: the TNP_{400} film was exposed to the electrolyte under no bias for 30 min duration prior to the test).

WILEY-VCH

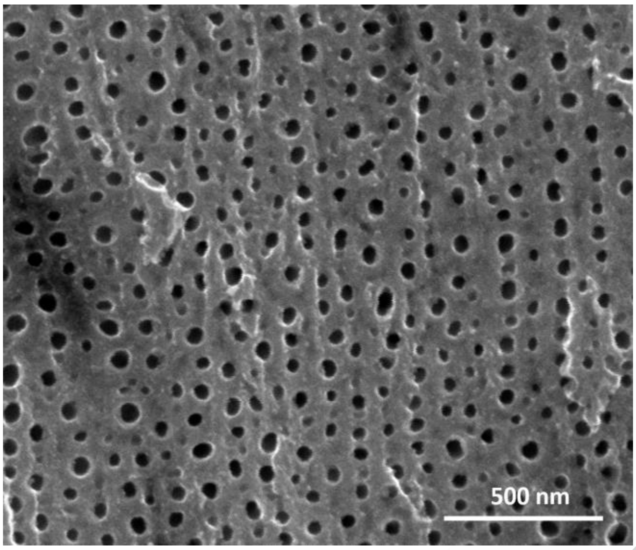

Figure S10. Top view FEG-SEM image of the TNP_{400} film after 48-hour stability test under illumination at the bias voltage of 0.8 V in 2.0 M KOH + PET electrolyte (NB: the TNP_{400} film was exposed to the electrolyte under no bias for 30 min duration prior to the test).

WILEY-VCH

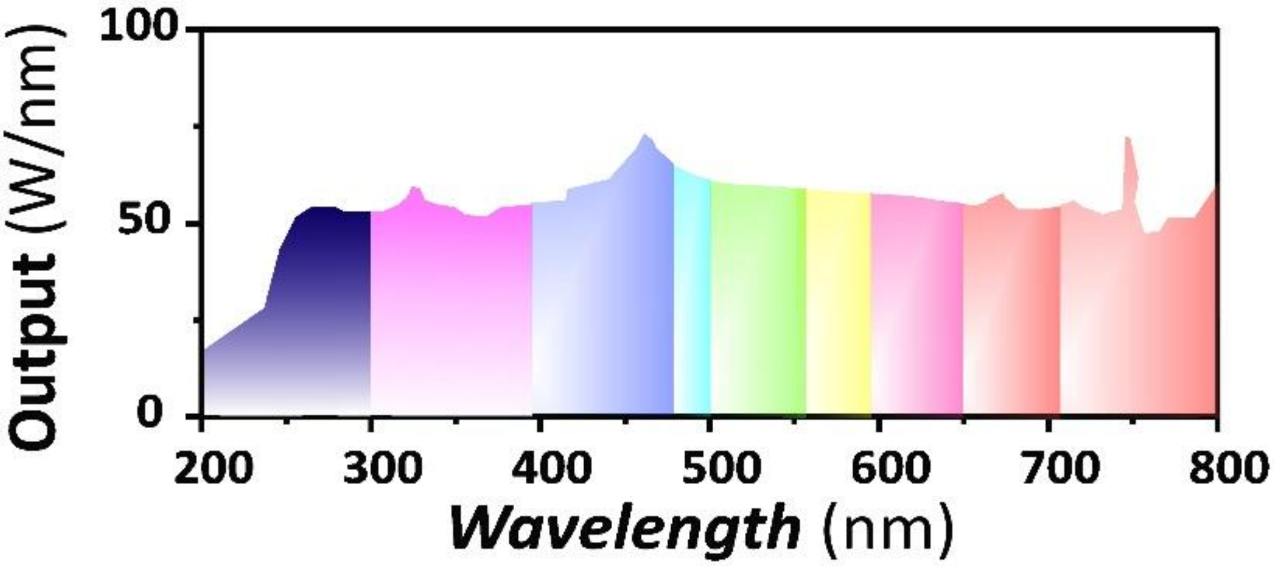

Figure S11. Emission spectrum of the Xenon lamp used in our study with an overall output power of 300 W.

WILEY

Table S1. A comparison of photocurrent density of different TiO_2-based systems in alkaline electrolyte conditions.

Photoanodes	Fabrication Method	Photocurrent density / Bias (mA cm^{-2})/ (V *vs* RHE)	Electrolyte /simulated solar light	Refs
TNPs thin films	Anodization and annealing	≈1.06 / 1.8	1 M KOH (pH = 13.6) (UV–Vis–NIR)	This work
TiO_2 thin films	Thermal oxidizing and Ar^+ ion irradiation	0.56 / –	1 M NaOH (AM 1.5G)	[1]
PDAP-3DTiO_2MSs/Ti[a]	Hydrothermal method	1.56 / 1.23	1 M NaOH (pH=13.6) (AM 1.5G)	[2]
FeNi/TiO_2	Spin-coating sol-gel precursor for TiO_2 and electrodeposition of FeNi	1.0 / –	0.1 M NaOH (pH=13) (AM 1.5G)	[3]
NiO/TiO_2	Reactive-ion sputtering and rapid thermal processing	1.68 / 1.23	0.1 M KOH (AM 1.5G)	[4]
TiO_2/S-doped TiO_2NWAs[b]	Hydrothermal sulfurization approach	1.24 / 1.23	1.0 M NaOH (AM 1.5G)	[5]
C/N co-doped TiO_2NWAs	Hydrothermal method, ion implantation	0.76 / 0.8[c]	1 M NaOH (pH=13.6) (AM 1.5 G, >420 nm)	[6]
Al−TiO_2NRAs	Hydrothermal method	0.75 / 1.23	0.1 M NaOH (AM 1.5G)	[7]
TiO_2NRAs	Hydrothermal procedure	0.978 / 1.23	1.0 M NaOH (pH=13.6) (AM 1.5G)	[8]
Gd−TiO_2 NRAs/FTO	Hydrothermal method	0.51 / 0.23[c]	1 M KOH (AM 1.5G)	[9]
Mixed-phase TiO_2 NTAs	Anodization of Ti foils	0.11 / 0.5[c]	1 M NaOH (AM 1.5G)	[10]
Au NPs/TiO_2NTAs[d]	Anodization and dip-coating	0.14 / 1.23	1 M KOH (>415 nm)	[11]

[a] MS, microspheres; [b] NWAs, nanowire arrays. [b] NRAs, nanorod arrays; [c] V vs. Ag/AgCl; [d] NTAs, nanotubes arrays.

WILEY-VCH

Table S2. Parameters of the equivalent circuit describing the electrolyte–electrode interface behavior of model TNP films.

Circuit components	Symbol	Units	TNP_{RT}	TNP_{400}	TNP_{900}
**Electrolyte resistance**	R_1	Ω	18.4 ± 1	4.1 ± 0.5	110.6 ± 3
**Double layer capacitance**	Q_2	$\mu F \cdot s^{(a-1)}$	25.9	73.3	25.8
**Factor**	a_2		0.89	0.35	0.50
**Charge transfer resistance**	R_2	Ω	6755 ± 50	948 ± 15	8436 ± 65
**TNP film capacitance**	Q_3	$\mu F \cdot s^{(a-1)}$	8.10	0.25	0.09
**Factor**	a_3		0.92	0.75	0.85
**TNP film resistance**	R_3	Ω	2930 ± 20	281 ± 8	482 ± 11

WILEY - VCH

Table S3. Estimation of charge carrier density, N_d, in the TNP films from Equation 3.

Factors	TNP_{RT}	TNP_{400}	TNP_{900}
**Slope, ($\frac{d\left(\frac{1}{C^2}\right)}{dE}$)**	6.53 ± 0.06	4.05 ± 0.05	8.87 ± 0.08
**Average dielectric constant**	20 (amorphous)	31 (anatase)	80 (rutile)
**Charge carrier density, N_d (cm^{-3})**	2.67×10^{20}	2.78×10^{20}	4.92×10^{19}

WILEY-VCH

Table S4. PEC–PET reformation performances of different catalysts.

Plastics	Catalyst	Electrolytes	Light source	**Bias** (V *vs* RHE)	**Current density** (mA cm^{-2})	**H_2 evolution** (μmol cm^{-2} h^{-1})	**Formate yield** (mmol L^{-1})	**Formate Faradaic efficiency (FE)** (%)	Refs
Pre-PET[a]	TNPs	2.0 M KOH	Xenon Lamp 300W	0.8[b]	2.34 ± 0.67	26.4 ± 0.4	1.68 ± 0.05	85 ± 9.0 in 3 h	This work
Pre-PET	Mo:BiVO4/NiCo-LDH	0.1 M KOH	HAL-320 solar simulator	–	2.3	–	0.5	≈ 85	[12]
Pre-PET	Ni-Pi/Fe_2O_3[c]	1.0 M NaOH	Xenon Lamp 300W	1.1	1.5	21.72	–	87	[13]
Pre-PET	Ti – Fe_2O_3/$Ni(OH)_x$	1.0 M KOH	AM 1.5G simulated solar irradiation	1.2	–	–	0.0277 [d]	95 in 30 h	[14]
Pre-PET	Fe_2O_3/$Ni(OH)_x$	1.0 M KOH	—	1.2	–	–	1.8[e]	≈100	[15]

[a] Pretreated PET, [b] two electrode PEC system, [c] Nickel phosphate (Ni-Pi) on α-Fe_2O_3, [d] μmol cm^{-2} h^{-1}, [e] 10C charge passed.

WILEY-VCH

References

[1] H. Wu, Z. Wang, S. Jin, X. Cao, F. Ren, L. Wu, Z. Xing, X. Wang, G. Cai, C. Jiang, *Int. J. Hydrogen Energy* **2018**, *43*, 6936.

[2] Y. Yu, N. Zhong, J. Zheng, S. Tang, X. Ye, T. Zeng, W. Yu, Z. He, S. Song, *Int. J. Hydrogen Energy* **2020**, *45*, 216.

[3] H. Li, H. Yang, Z. Li, X. Wang, X. Liu, S. Bandaru, X. Zhang, *Electrochim. Acta* **2021**, *387*, 138533.

[4] T. T. Nguyen, M. Patel, S. Kim, V. A. Dao, J. Kim, *ACS Appl. Mater. Interfaces* **2021**, *13*, 10181.

[5] Z. Li, Y. Li, C. Wang, M. Zhang, L. Huang, J. Lai, L. Wang, J. Li, X. Jin, W. Yang, *J. Alloys Compd*. **2021**, *858*, 158375.

[6] X. Song, W. Li, D. He, H. Wu, Z. Ke, C. Jiang, G. Wang, X. Xiao, *Adv. Energy Mater*. **2018**, *8*, 1800165.

[7] R. Lu, Y. Wei, C. Chen, T. Wu, *J. Alloys Compd*. **2019**, *790*, 99.

[8] H. Huang, X. Hou, J. Xiao, L. Zhao, Q. Huang, H. Chen, Y. Li, *Catal. Today* **2019**, *330*, 189.

[9] A. Ahmad, G. Yerlikaya, R. Zia ur, H. Paksoy, G. Kardaş, *Int. J. Hydrogen Energy* **2020**, *45*, 2709.

[10] R. Yalavarthi, A. Naldoni, Š. Kment, L. Mascaretti, H. Kmentová, O. Tomanec, P. Schmuki, R. Zbořil, *Catalysts* **2019**, *9*, 204.

[11] S. Y. Moon, H. C. Song, E. H. Gwag, I. Nedrygailov, C. Lee, J. J. Kim, W. H. Doh, J. Y. Park, *Nanoscale* **2018**, *10*, 22180.

[12] F. Kang, Q. Wang, D. Du, L. Wu, D. W. F. Cheung, J. Luo, *Angew. Chem. - Int. Ed.* **2025**, *64*, e202417648.

[13] B. Zhang, H. Zhang, Y. Pan, J. Shao, X. Wang, Y. Jiang, X. Xu, S. Chu, *J. Chem. Eng*. **2023**, *462*, 142247.

[14] X. Li, J. Wang, M. Sun, X. Qian, Y. Zhao, *J. Energy Chem*. **2023**, *78*, 487.

[15] X. Li, J. Wang, T. Zhang, T. Wang, Y. Zhao, *ACS Sustain. Chem. Eng.* **2022**, *10*, 9546.

WILEY-VCH
